# Abrogation of LRRK2 dependent Rab10 phosphorylation with TLR4 activation and alterations in evoked cytokine release in immune cells

**DOI:** 10.1016/j.neuint.2021.105070

**Published:** 2021-05-15

**Authors:** Iqra Nazish, Charles Arber, Thomas M. Piers, Thomas T. Warner, John A. Hardy, Patrick A. Lewis, Jennifer M. Pocock, Rina Bandopadhyay

**Affiliations:** a Reta Lila Weston Institute and Department of Clinical and Movement Neuroscience, UCL Queen Square Institute of Neurology, 1 Wakefield Street, WC1N 1PJ, UK; b Department of Neurodegenerative Diseases, UCL Queen Square Institute of Neurology, 1 Wakefield Street, WC1N 1PJ, UK; c Department of Neuroinflammation, UCL Queen Square Institute of Neurology, 1 Wakefield Street, WC1N 1PJ, UK; d Queen Square Genomics, UCL Dementia Research Institute, Wing 1.2 Cruciform Building, Gower Street, London, WC1E 6BT, UK; e Royal Veterinary College, Royal College Street, London, NW1 0TU, UK

**Keywords:** LRRK2 phosphorylation, RAW264.7, LPS, Zymosan, Rab10 phosphorylation, MAPK, TAK242, Sparstolonin B, Cytokine release, IPS-Macrophage

## Abstract

LRRK2 protein is expressed prominently in immune cells, cell types whose contribution to LRRK2-associated genetic Parkinson’s disease (PD) is increasingly being recognised. We investigated the effect of inflammatory stimuli using RAW264.7 murine macrophage cells as model systems. A detailed time course of TLR2 and TLR4 stimulation was investigated through measuring LRRK2 phosphorylation at its specific phospho-sites, and Rab8 and Rab10 phosphorylation together with cytokine release following treatment with LPS and zymosan. LRRK2 phosphorylation at Ser935, Ser955 and Ser973 was increased significantly over untreated conditions at 4–24h in both WT-LRRK2 and T1348N-LRRK2 cell lines to similar extents although levels of Ser910 phosphorylation were maintained at higher levels throughout. Importantly we demonstrate that LPS stimulation significantly decreased phospho-Rab10 but not phospho-Rab8 levels over 4–24h in both WT-LRRK2 and T1348N-LRRK2 cell lines. The dephosphorylation of Rab10 was not attributed to its specific phosphatase, PPM1H as the levels remained unaltered with LPS treatment. MAPK phosphorylation occurred prior to LRRK2 phosphorylation which was validated by blocking TLR4 and TLR2 receptors with TAK242 or Sparstolonin B respectively. A significant decrease in basal level of TNFα release was noted in both T1348N-LRRK2 and KO-LRRK2 cell lines at 48h compared to WT-LRRK2 cell line, however LPS and zymosan treatment did not cause any significant alteration in the TNFα and IL-6 release between the three cell lines. In contrast, LPS and zymosan caused significantly lower IL-10 release in T1348N-LRRK2 and KO-LRRK2 cell lines. A significant decrease in phospho-Rab10 levels was also confirmed in human IPS-derived macrophages with TLR4 activation. Our data demonstrates for the first time that LRRK2-dependent Rab10 phosphorylation is modulated by LPS stimulation, and that cytokine release may be influenced by the status of LRRK2. These data provide further insights into the function of LRRK2 in immune response, and has relevance for understanding cellular dysfunctions when developing LRRK2-based inhibitors for clinical treatment.

## 1 Introduction

Pathogenic, autosomal-dominant missense mutations in the *leucine-rich repeat kinase 2* (*LRRK2*) gene on chromosome 12 are the most frequent cause of late-onset PD (Healy et al., 2008; Paisan-Ruiz et al., 2004; Zimprich et al., 2004) and non-coding variation at the *LRRK2* locus has been identified as being associated with life time risk of developing idiopathic PD (Haugarvoll and Wszolek, 2009). *LRRK2* PD patients present similar clinical features to those observed in idiopathic forms, Neuropathologically, the majority (~90%) of LRRK2-mutated cases display alpha-synuclein positive Lewy bodies (LBs) and Lewy neurites (LNs) at post-mortem, although a subset of patients demonstrate pleomorphic pathologies featuring tau deposits, TDP-43 and ubiquitin only inclusions. However, loss of dopaminergic neurons in the substantia nigra is a common link amongst all *LRRK2* mutation carriers (Wider et al., 2010).

LRRK2 protein is a large, multidomain protein comprising of two enzymatic domains: a ROC-COR domain (GTPase domain) and kinase domain at its core. The enzymatic core is flanked by several proteinprotein interaction domains thought to play roles in signalling and scaffolding functions (Liao and Hoang, 2018). Pathogenic mutations in *LRRK2,* associated with PD, cluster within the ROC-COR and the kinase domains, implicating the enzymatic activity of this protein as being key to its role in PD. The G2019S mutation located within the kinase domain is the most common familial PD mutation identified to date (Lesage et al., 2006; Ozelius et al., 2006; Tolosa et al., 2020) and is also present in sporadic PD patients (Healy et al., 2008). *In vitro* studies have shown that pathogenic LRRK2 mutations result in altered kinase activity, and that this may lead to increased neuronal toxicity (Greggio et al., 2006; Luzon-Toro et al., 2007; West et al., 2005). Notably, the toxic effects of mutant LRRK2 overexpression can be attenuated by LRRK2 kinase inhibitors in animal models of PD (Lee et al., 2010). The GTPase domain contributes to the regulation of LRRK2’s kinase activity and also in its dimerization (Nguyen and Moore, 2017). Familial mutations located in the ROC-COR tandem domain increase GTP binding and R1441 C/G/H and Y1699C mutations all exhibit decreased GTP hydrolysis when compared to WT-LRRK2 (Biosa et al., 2013; West et al., 2007; Xiong et al., 2010). The T1348N-LRRK2 is an artificial P-Loop null mutation that disrupts GTP binding but a side effect of the mutation causes a reduction in dimerization and compromises protein stability (Biosa et al., 2013; Ito et al., 2016). Additionally, autophosphorylation sites within the ROC-COR domain may also regulate kinase activity (Nguyen and Moore, 2017). As the kinase activity steers toxicity and pathology of LRRK2, consequently Phase II clinical trials are in progress through Denali Therapeutics NCT04056689) for small molecule kinase inhibitors, and LRRK2 antisense technology trials through Biogen (NCT03976349).

Because of its complex protein structure, LRRK2 is involved in a number of biological processes and signalling pathways including immune cell functionality (Harvey and Outeiro, 2019; Wallings et al., 2015). Interestingly, polymorphisms at the *LRRK2* locus have been associated with heightened risk of developing Inflammatory Bowel Disease, an autoimmune disorder, and multibacillary leprosy (Barrett et al., 2008; Zhang et al., 2009). Importantly, LRRK2 is a member of the RIP kinase family, members of which respond to cellular stress (Meylan and Tschopp, 2005), and its expression is increased upon pro-inflammatory stimuli in immune cells (Daher et al., 2014; Hakimi et al., 2011). Full length LRRK2 is expressed in peripheral blood mononuclear cells (PBMCs), monocytes, B- and T cells, and LRRK2 gene expression is upregulated in response to microbial structures (Hakimi et al., 2011) and may play a role in monocyte maturation (Gillardon et al., 2012; Thevenet et al., 2011). Lipopolysaccharide (LPS) stimulation in mice overexpressing the R1441G mutation results in an increased secretion of pro-inflammatory cytokines, leading to neurotoxicity (Gillardon et al., 2012). Interferon-gamma (IFNγ) can mediate induction of LRRK2 in acute monocytic leukemia THP-1 cells and human peripheral blood monocytes which is dependent on the ERK pathway (Kuss et al., 2014). In addition, LRRK2 inhibitor treatment of PBMCs from PD patients results in dephosphorylation of LRRK2 constitutive phosphorylation (Perera et al., 2016). In a recent study, LRRK2 levels in peripheral immune cells were shown to be increased in PD patients (Cook et al., 2017), suggesting that LRRK2 immune cell expression could act as a disease biomarker for this disorder.

LRRK2 protein has a number of phospho-sites that can either be autophosphorylated or constitutively phosphorylated by other kinases (De Wit et al., 2018). Activation of toll-like receptors 2 and 4 (TLR2 and TRL4) leads to marked phosphorylation of LRRK2 at Ser910 and Ser935 residues resulting in recruitment of 14–3–3 proteins and re-localisation of LRRK2 in types of myeloid cells (Dzamko et al., 2012; Nichols et al., 2010; Schapansky et al., 2014). The phosphorylation increases at Ser935 and Ser910 of LRRK2 with TLR agonists being independent of LRRK2 kinase activity (Dzamko et al., 2012). In addition, there are two additional phospho-residues at Ser955 and Ser973 in LRRK2 and phosphorylation of all four of these phospho-residues are sensitive to LRRK2 kinase inhibition at the basal level. Upon phosphorylation of Ser910/935, LRRK2 translocates from cytosol to the membrane. Membrane associated LRRK2 co-localises to autophagosomal membranes following either TLR4 stimulation or mTOR inhibition with rapamycin. However, the absence of LRRK2 activity in BV2 murine microglia or murine RAW264.7 macrophages had no effect on phagocytosis (Schapansky et al., 2014). Using quantitative mass spectrometry, Sheng et al. (2012) reported that LRRK2 phosphorylates itself at Ser1292 site both *in vivo* and *in vitro*. More recently, LRRK2 has been shown to directly phosphorylate a subset of Rab GTPases, including Rab8, Rab10 and Rab29 (also known as Rab7L1) (Steger et al., 2016). Importantly, this supports a link between LRRK2 and the regulation of vesicle trafficking (Ebanks et al., 2019), a link that may have important implications for the transport of inflammation induced cytokines including TNFα.

In this study we have investigated the phospho-regulation of LRRK2 and its substrates following TLR stimulation using murine RAW264.7 cells as a model for LRRK2, taking advantage of genome edited RAW264.7 cells lacking LRRK2 or carrying a mutation T1348N that ablates LRRK2 GTP binding. Additionally we have verified part of the data in human IPS-Macrophage (iPS-Mac) cells. The specificity of TLR activation was investigated using antagonists/inhibitors of TLR2 (Sparstolonin B) and TLR4 (TAK242), and finally we measured cytokine release, namely TNFα, IL-6 and IL-10 in the above-mentioned cell lines with TLR activation.

## 2 Materials and methods

### 2.1 Cell culture

Three RAW264.7 murine macrophage cell lines were obtained from American Type Culture Collection: wild-type (#SC-6003), T1348N-LRRK2 (#SC-6005), and KO-LRRK2 (#SC-6004). Both the T1348N and KO-LRRK2 RAW264.7 cell lines are homozygous. Cells were incubated in a 100% relative humidified incubator (95% air, 5% CO_2_) at 37°C and were grown in Dulbecco’s Modified Eagle Medium (DMEM) (Life Technologies #41965–039) (+4.5 g/L D-glucose, +L-glutamine, -pyruvate) supplemented with 10% heat-inactivated foetal bovine serum (FBS) (Thermofisher #10500) and 1% penicillin, streptomycin and Amphotericin B (Thermofisher #15240062). The culture medium was refreshed every 2–3 days of culture. For treatments, 200,000 cells were seeded in each 6 well plates. Following treatments, cells were collected in modified RIPA buffer [25 mM Tris, 50 mM NaCl, 1 mM EDTA, 0.5% NP-40 and 0.25% Na deoxycholate and protease (Phos-STOP, Roche) and phosphatase inhibitors (cOmplete Mini protease inhibitor cocktail, Roche)]. Protein lysates were collected and centrifuged at 14,000×g for 15min to pellet cell debris and supernatants collected and stored on ice or at −20 °C for further use.

iPS-Mac culture: iPS-Macs were grown from a human control cell line obtained from EBiSC, BIONi010-C and plated at 500k/well of a 6 wlll plate as described in Garcia-Reitboeck et al. (2018). Three separate platings of this cell line were used in this study. The iPS-Macs were treated with LPS (100 ng/ml) and zymosan (200 μg/ml) for 4 h and cells collected in modified RIPA buffer and processed similarly as described for Raw264.7 cells.

### 2.2 Protein assay

Protein levels were measured using the BIO-RAD-DC protein assay kit as per manufacturer’s instructions using BSA as standard.

### 2.3 LPS and zymosan treatment protocol

Required final concentrations of lipopolysaccharide, (LPS, 100 ng/ ml, Salmonella serotype enteridis; Sigma L7770) and zymosan 200 μg/ml (Sigma Z4250) were dissolved in cell media prior to experimentation.

### 2.4 TAK242 and Sparstolonin B treatment

TAK242 was obtained from Tocris (cat no: 243984-11-4) and Sparstolonin B was obtained from Sigma (Cat no: SML1767). Stock solutions were dissolved in DMSO and the required concentrations of 1 μM for TAK242 and 50 μM Sparstolonin B were added to the required medium. The doses of TAK242 and Sparstolonin B were chosen based on their use in previously published works (Liang et al., 2011; Matsunaga et al., 2011). Small and equivalent amounts of DMSO were also added to the control cultures. Cells were pre-treated with inhibitors for 45min before LPS and zymosan treatment was started for 30min and 4h. Treatments with inhibitors only were also included in the study.

### 2.5 Immunoblots

Frozen cell lysates extracted with RIPA buffer were thawed on ice and 20 μg/μl of protein sample was loaded on Criterion TGX Precast 18-well 4%–20% Midi protein gel (Bio-Rad). Standard protocol was used for immunoblotting and has been described in details by our group in a previous publication (Mamais et al., 2013). The antibodies used are listed in (Table 1).

### 2.6 QRT-PCR

RNA was extracted using T RIzol reagent using manufacturer’ s instructions (Thermo Fisher).

To determine the RNA concentration and purity, a Nanodrop spectrophotometer (Thermoscientific) was used. 2 μg of RNA was then reverse transcribed using SuperscriptIV (Thermo Fisher) using random hexamers. Power SYBR green master mix (Thermo Fisher) was used for QRT-PCR using Mx3000P System (Agilent). Data were generated using the ΔΔCT methods. Results were normalised to the reference genes GAPDH and B2M and presented relative to one control wild-type sample (Pfaffl, 2001). Details of primer pairs used are as follows (annealing temperature 60°C for all):

   GAPDH:   F:   5’-GCATCTTCTTGTGCAGTGCC,   R:   5’-TCACACCCATCACAAACATG

   B2M:   F:   5’-CACTGAATTCACCCCACT,   R:   5’-TGTCTCCATCCCAGTAGAC

   TLR2:   F:   5’-TGTAGGTGATCTTGTTGAAA,   R:   5’-TCAGACAAAGCGTCAAAT

   TLR4:   F:   5’-AATGAGAATGATGAAGGAA,   R:   5’-CTGAATGACAAGACTACA

### 2.7 Cell counting

Cells at a density of 20,000 were grown on glass coverslips in 24-well plates and were treated with the above-mentioned doses of LPS or zymosan for 24h. To determine the number of live and dead cells following TLR4 or TLR2 stimulation, cellular nuclei were stained with propidium iodide (PI, 5 μg/ml final concentration) to give the number of dead cells, and Hoechst 33342 (17.5 μM final concentration) for 15 min at 37 °C for the total number of cells. Cells were viewed using a Zeiss Axioskop 2 fluorescence microscope with 20x Neofluor objective (Oberkochen, Germany). Five fields were randomly chosen to image per treatment per experiment at 20x magnification and all the living and dead cells in those fields were counted. The data was then represented in histograms as the percentage of dead cells.

### 2.8 Enzyme linked immunosorbent assay (ELISA)

The three cell lines, WT-LRRK2, T1348N-LRRK2 and KO-LRRK2 RAW264.7 were treated with LPS and zymosan for 24 and 48 h. Media supernatants were then collected after 24 and 48 h which were then used to carry out ELISA assays. ELISA assays were performed using mouse tumor-necrosis factor alpha (TNFα), interleukin-6 (IL-6) and Interleukin-10 (IL-10) Quantikine ELISA kits (R&D Systems) according to the manufacturer’s instructions. Samples, run in triplicates, were normalised against ELISA kit controls. The values were then normalised over concentrations of total protein.

### 2.9 Statistics

One-way analysis of variance (ANOVA) with Tukey’s post-hoc test was used to compare the difference between non-stimulated cells and cells stimulated with LPS or zymosan between the means of three or more independent groups, e.g. when determining any significant difference between the three cell lines, phospho-sites and time-points. Twotailed student’ s T-test was used to determine the significant difference between the means of two groups. All immunoblot data display replicates from one experiment. Statistics has been performed using all the data points for all experiments.

## 3 Results

### 3.1 Phosphorylation of Ser910, p935, p955 and p973 LRRK2, Rab8 and Rab10 in WT-LRRK2, T1348N-LRRK2 and KO-LRRK2 RAW264.7 cells

**Fig. 1:**
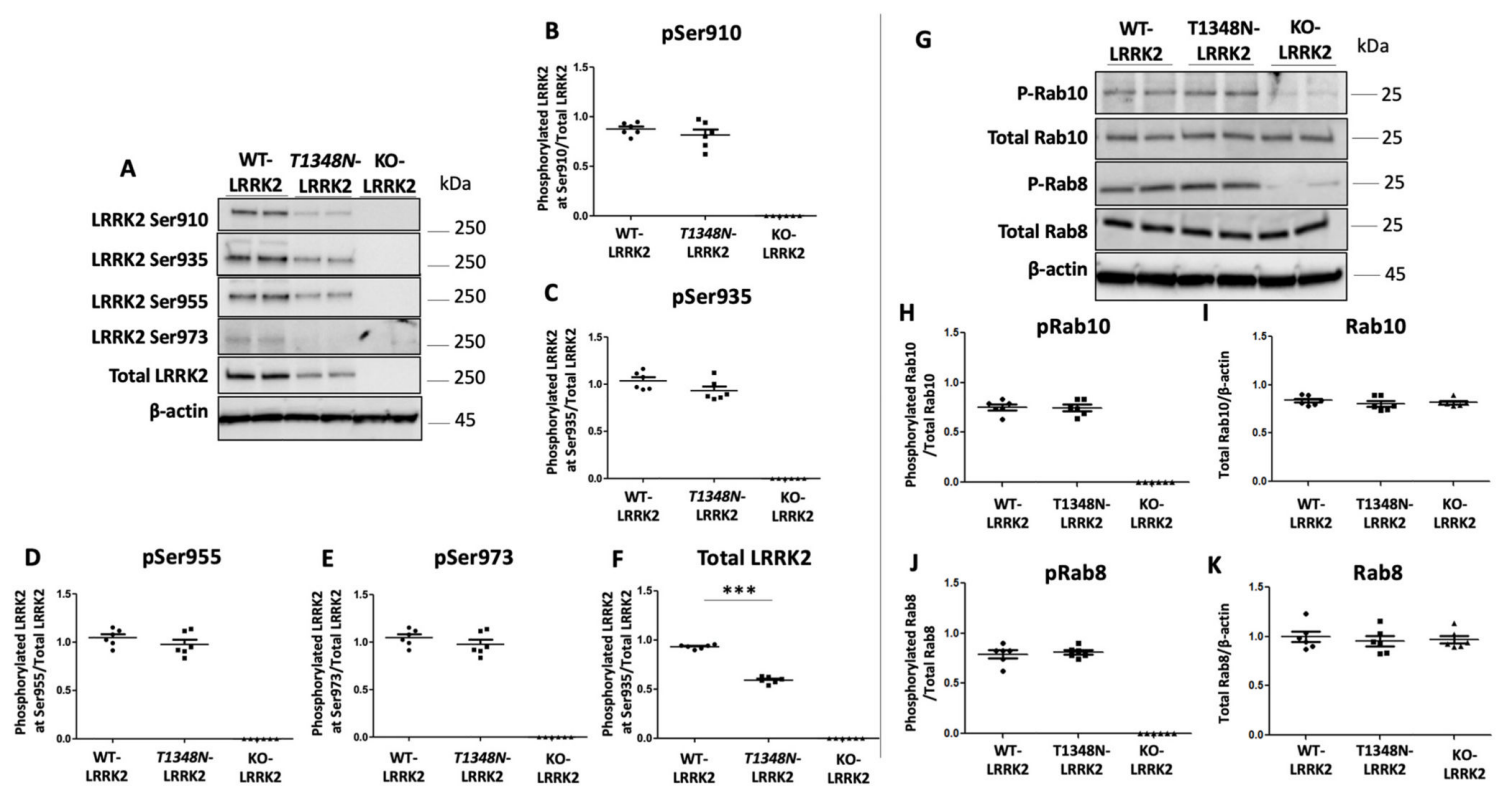
Levels of phosphorylated Ser935 in WT-LRRK2 and T1348N-LRRK2 and phosphorylated Rab8 and Rab10 in KO-LRRK2 RAW264.7 macrophage cells. Immunoblots from WT-LRRK2, T1348N-LRRK2 and KO-LRRK2 cells of basal phospho-Ser935 levels (A, B), phospho-Ser910 levels (A, C), phospho-Ser955 levels (A, D), phospho-Ser973 levels (A, E) and total LRRK2 levels (A, F). Immunoblots from WT-LRRK2, T1348N-LRRK2 and KO-LRRK2 of phospho-Rab10 levels (G, H), total Rab10 levels (G,I), phospho-Rab8 levels (G, J) and total Rab8 levels (G, K). Values represent the mean ± S.E.M. of 3 independent experiments (with internal duplicates in each experiment). Statistical significance was determined using Two-tailed students T-test. *** denote p < 0.001.

We investigated baseline levels of phosphorylation of LRRK2 Ser910, Ser935, Ser955 and Ser973, Rab8 T72 and Rab10 T73 in the three cell lines as these are readouts of LRRK2 kinase activity. This was accomplished using immunoblot analysis with phosphorylation specific antibodies (Fig. 1). The T1348N-LRRK2 and the KO-LRRK2 RAW264.7 are homozygous cell lines which were produced by gene editing technology that yields endogenous levels of proteins which circumvents the artefacts arising in cell lines that are associated with overexpression.

There was no LRRK2 or phospho-LRRK2 (Ser910, Ser935, Ser955, Ser973) expression in the KO-LRRK2 cell line. Comparison of phosphorylation levels of Ser935 between WT-LRRK2 and T1348N-LRRK2 cells revealed no significant alteration in T1348N-LRRK2 compared with WT-LRRK2 cells (Fig 1A, B–E). Total LRRK2 levels were significantly lower in T1348N cell line. (Fig. 1A, F). There was no detectable phosphorylated Rab8 (pRab8) or phosphorylated Rab10 (pRab10) in KO-LRRK2 cells compared with WT-LRRK2 cells (Fig. 1G) whilst total Rab8 and Rab10 were similar between WT-LRRK2 and KO-LRRK2 cells (Fig. 1H, I). These data offer further verification that Rab8 and Rab10 are indeed authentic substrates of LRRK2 phosphorylation, and that LRRK2 is the primary kinase responsible for phosphorylating these proteins at residues T72 and T73 respectively.

### 3.2 Stimulation of TLR2 and TLR4 enhances Ser935-LRRK2 and Ser955-LRRK2 phosphorylation in WT-LRRK2 and T1348N-LRRK2 RAW264.7 cells

**Fig. 2:**
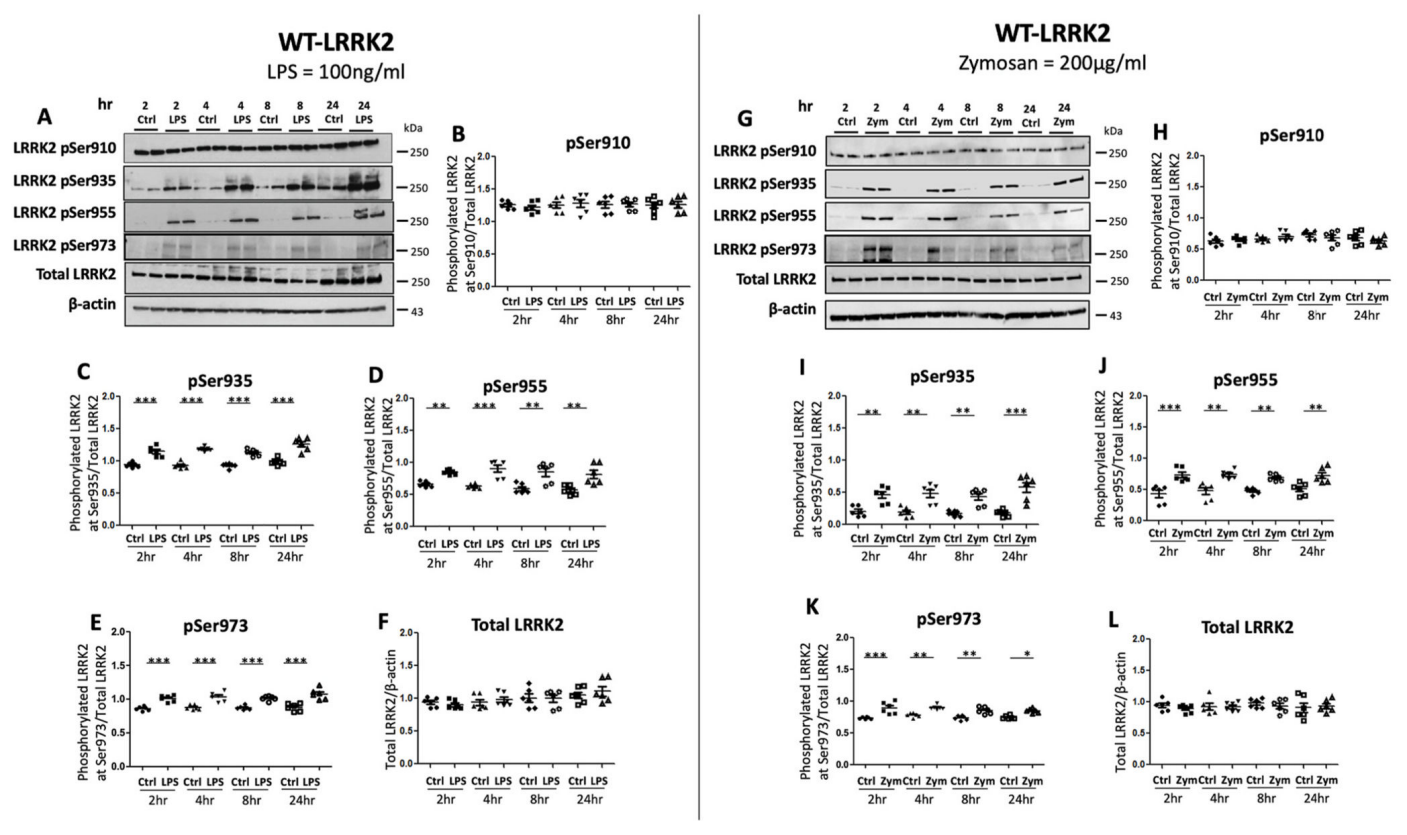
Time course of upregulation of LRRK2 Phosphorylation with LPS and zymosan in WT-LRRK2 RAW264.7 macrophage cells. WT-LRRK2 RAW264.7 macrophage cells were treated with 100 ng/ml LPS and zymosan (200 μg/ml) for 2h, 4h, 8h and 24h before cell pellets were subjected to immunoblotting with indicated antibodies. Controls contain media only. Blots were probed with LRRK2 phosphorylation specific antibodies, Ser910, Ser935 and Ser955 and Ser973, as well as total LRRK2 with LPS treatment (A) and corresponding quantifications (B-F) and with zymosan (G) and corresponding quantifications (H-L). Values represent the mean ± S.E.M. of 3 independent experiments (with internal duplicates in each experiment). Statistical analysis carried out by repeated measures one-way ANOVA with Tukey’s post-hoc test. ** and *** denotes statistical differences from control at p < 0.01 and p < 0.001 respectively.

LRRK2 phosphorylation at Ser910, Ser935, and Ser955 and Ser973 phospho-residues were assessed following LPS (100 ng/ml, TLR4 agonist) and zymosan (200 μg/ml, TLR2 agonist) at 2h, 4h, 8h and 24h time points in WT-LRRK2 (Fig. 2) and T1348N-LRRK2 RAW264.7 cell lines (Fig. S1) using immunoblots. There was a high level of basal phosphorylation at Ser910 in untreated conditions with no further significant increases observed with LPS treatment at all-time points tested in both cell lines (Fig. 2B and S1B). Immunoblots using phospho-site specific antibodies for Ser935, Ser955, Ser973 residues showed significant upregulation of phosphorylation at all these sites at 2 h treatment with LPS, which was maintained at 4h, 8h and 24h in both WT-LRRK2 and T1348N-LRRK2 cell lines (Fig. 2C–E and Fig. S1C–E). Total LRRK2 levels remained stable following LPS treatment for all the time points tested (Fig. 2F and Fig. S1F).

To investigate how zymosan affects LRRK2 phosphorylation at Ser910, Ser935, Ser955 and Ser973 residues of LRRK2, both cell lines were treated with 200 μg/ml of zymosan for 2h, 4h, 8h and 24h. Similar to that observed with LPS treatment, basal phosphorylation levels for Ser910 residue remained constant with no further upregulation seen with zymosan treatment at all time-points tested in both WT-LRRK2 (Fig. 2G,H) and T1348N-LRRK2 lines (Fig. S1G,H). However, significant upregulation in phosphorylation was observed at Ser935, Ser955 and Ser973 with zymosan residues at all time-points tested (Fig. 2I–K and Fig. S1I–K) although the extent of the increased phosphorylation was slightly lower compared with LPS treatment. Overall, the level of phosphorylation at LRRK2 Ser973 residue was lower in both basal levels and when stimulated with either LPS or zymosan compared to Ser910, Ser935 and Ser955. This however could reflect the lower specificity of the Ser973 antibody.

### 3.3 Stimulation of TLR4 but not TLR2 significantly reduces phosphorylation of Rab10 but not Rab8 in WT-LRRK2 and T1348N-LRRK2 cells

**Fig. 3:**
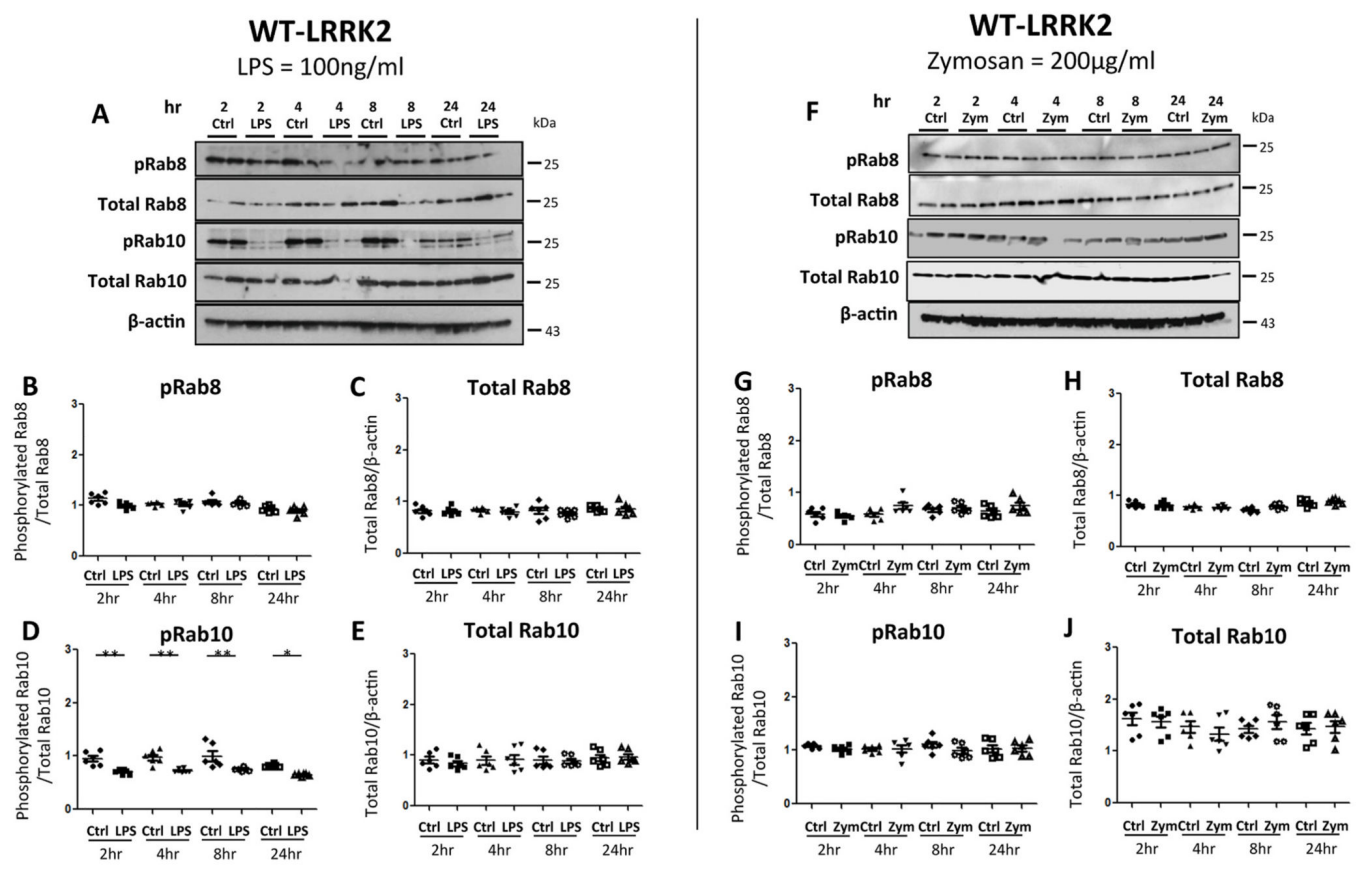
Rab8 and Rab10 phosphorylation with LPS and zymosan treatments in WT-LRRK2 RAW264.7 macrophage cells. WT-LRRK2 RAW264.7 macrophage cells were treated with 100 ng/ml LPS and zymosan (200 μg/ml) for 2h, 4h, 8h and 24h before cell pellets were subjected to immunoblotting with indicated antibodies. Controls contain media only. Blots were probed with Phospho-Rab8, Rab8, Phospho-Rab10 and Rab10 with LPS treatment (A) and corresponding quantifications (B-E) and with zymosan (F) and corresponding quantifications (G-J). Values represent the mean ± S.E.M. of 3 independent experiments (with internal duplicates in each experiment). Statistical significance measured with one-way ANOVA with Tukey’s post-hoc test. *, ** and *** different from control at p < 0.05, 0.01 and 0.001, respectively.

In order to investigate whether Rab8 or Rab10 phosphorylation was affected by LPS or zymosan treatment in the WT-LRRK2 and T1348N-LRRK2 cell lines, we treated cell lines with LPS (100 ng/ml) and zymosan (200 μg/ml) and examined the phosphorylation of Rab8 and Rab10 with phospho-specific antibodies at 2h-24h time points as before. Total Rab8 and Rab10 levels did not change with LPS treatment (Fig 3C, E and Fig. S2C,E). There was no change in Rab8 phosphorylation at all time-points tested with either LPS or zymosan treatment in WT-LRRK2 (Fig. 3B,G) and T1348N-LRRK2 cells (Fig. S2B,E). In contrast, phosphorylation of Rab10 in WT-LRRK2 (Fig. 3D) and T1348N-LRRK2 (Fig. S2D) cells was significantly decreased following LPS stimulation from 2h to 24h, whilst zymosan induced no decrease in phosphorylation of Rab10 in WT-LRRK2 and T1348N-LRRK2 cells (Fig. 3I and Fig. S2I).

### 3.4 PPM1H levels remain unaltered in WT-LRRK2 and T1348N-LRRK2 cell lines treated with LPS

In order to establish whether the levels of protein-phosphatse 1H (PPM1H) are altered as a result of LPS treatment on RAW264.7 cells, we treated the two cell lines with LPS for 2hr, 4hr, 8hr and 24hr after which cell extracts were subjected to immunoblots with PPM1H and β-actin. There was no alteration in the levels of PPM1H at any of the treatment time-points (Fig. S3).

### 3.5 Phosphorylation of MAPK occurs in WT-LRRK2, T1348N-LRRK2 and KO-LRRK2 cell lines treated with LPS or zymosan

**Fig. 4:**
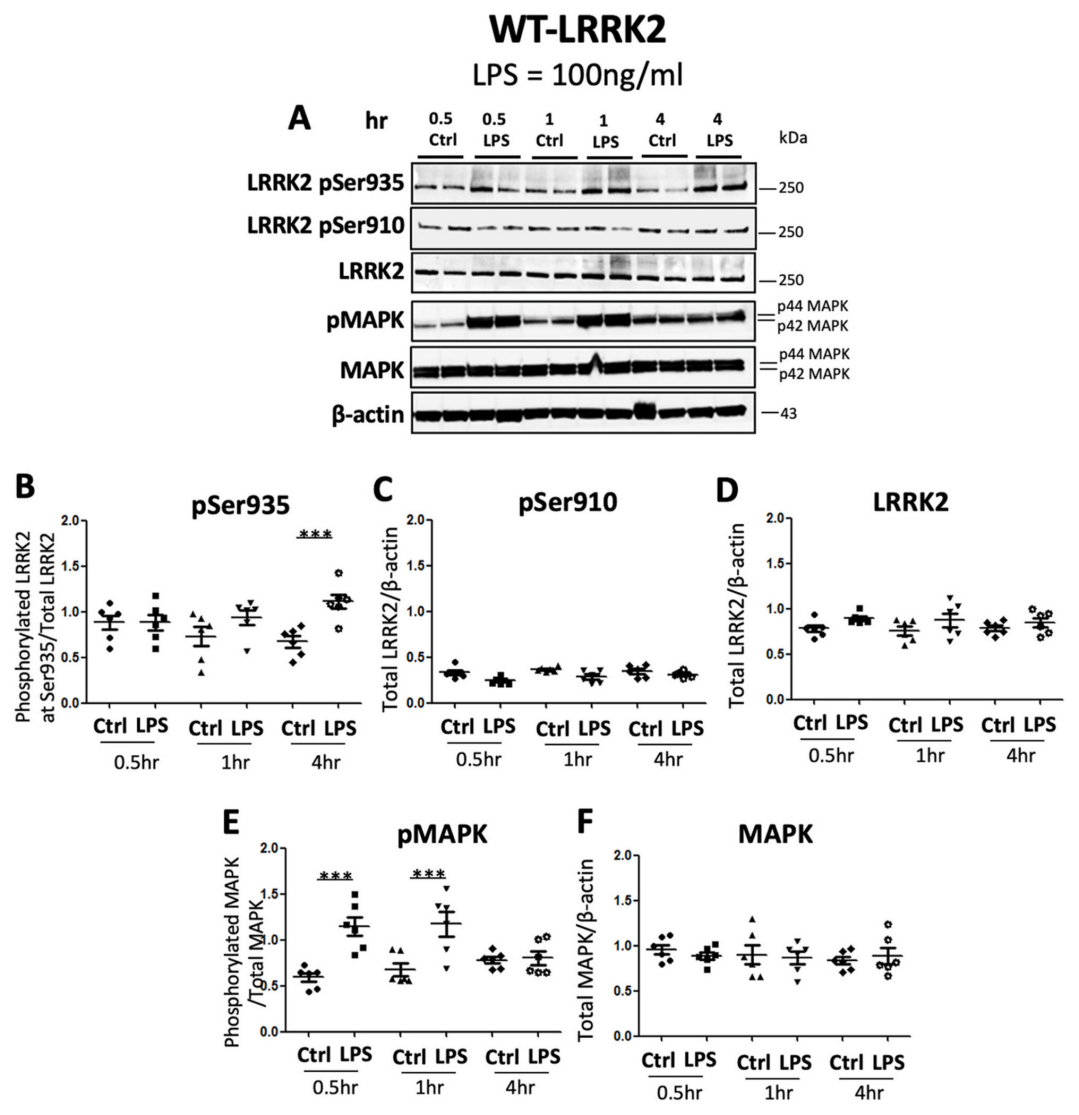
MAPK phosphorylation occurs prior to LRRK2 phosphorylation with LPS treatment in WT-LRRK2 RAW264.7 macrophage cells. WT-LRRK2 RAW264.7 macrophage cells were subjected to LPS (100 ng/ml) treatment and cell pellets were collected after 30min, 1h and 4h treatment timepoints and subjected to immunoblotting procedure with the indicated antibodies. Controls contain media only. Blots were probed with LRRK2 phosphorylation antibodies Phospho-Ser935, Phospho-Ser910, Phospho-p44/p42 MAPK (pMAPK), as well as total LRRK2 and MAPK (A) and corresponding quantifications (B-F). Values represent the mean ± S.E.M of 3 independent experiments (with internal duplicates in each experiment). Statistical significance carried out by repeated measures one-way ANOVA with Tukey’s post-hoc test. *** denotes statistical significance compared to controls at p < 0.01 and 0.001, respectively.

TLR stimulation leads to activation of the MAPK pathway resulting in phosphorylation of MAPK (Kawasaki and Kawai, 2014).Therefore in order to test whether MAPK phosphorylation occurs earlier than LRRK2 phosphorylation we performed a time course of TLR-stimulation with LPS or zymosan, we treated cells with LPS or zymosan for 30min, 1h or 4h. As expected, significant phosphorylation was observed at Ser935 LRRK2 residue at 4h time point in WT-LRRK2 (Fig. 4B) and T1348N-LRRK2 cells (Fig.4B), while there was no significant upregulation in Ser910 at any time-point (Fig. 4C, Fig. S4C). MAPK phosphorylation increased significantly at 30min and 1h but not at 4h in all three cell lines, WT-LRRK2, (Fig. 4E), T1348N-LRRK2, (Fig. S4E) and KO-LRRK2, (Fig. S4H).

### 3.6 Effect of TLR4 and TLR2 inhibitors (TAK242 and Sparstolonin B respectively) on LRRK2 Ser935 and MAPK phosphorylation in WT-LRRK2 and T1348N-LRRK2 cells

**Fig. 5:**
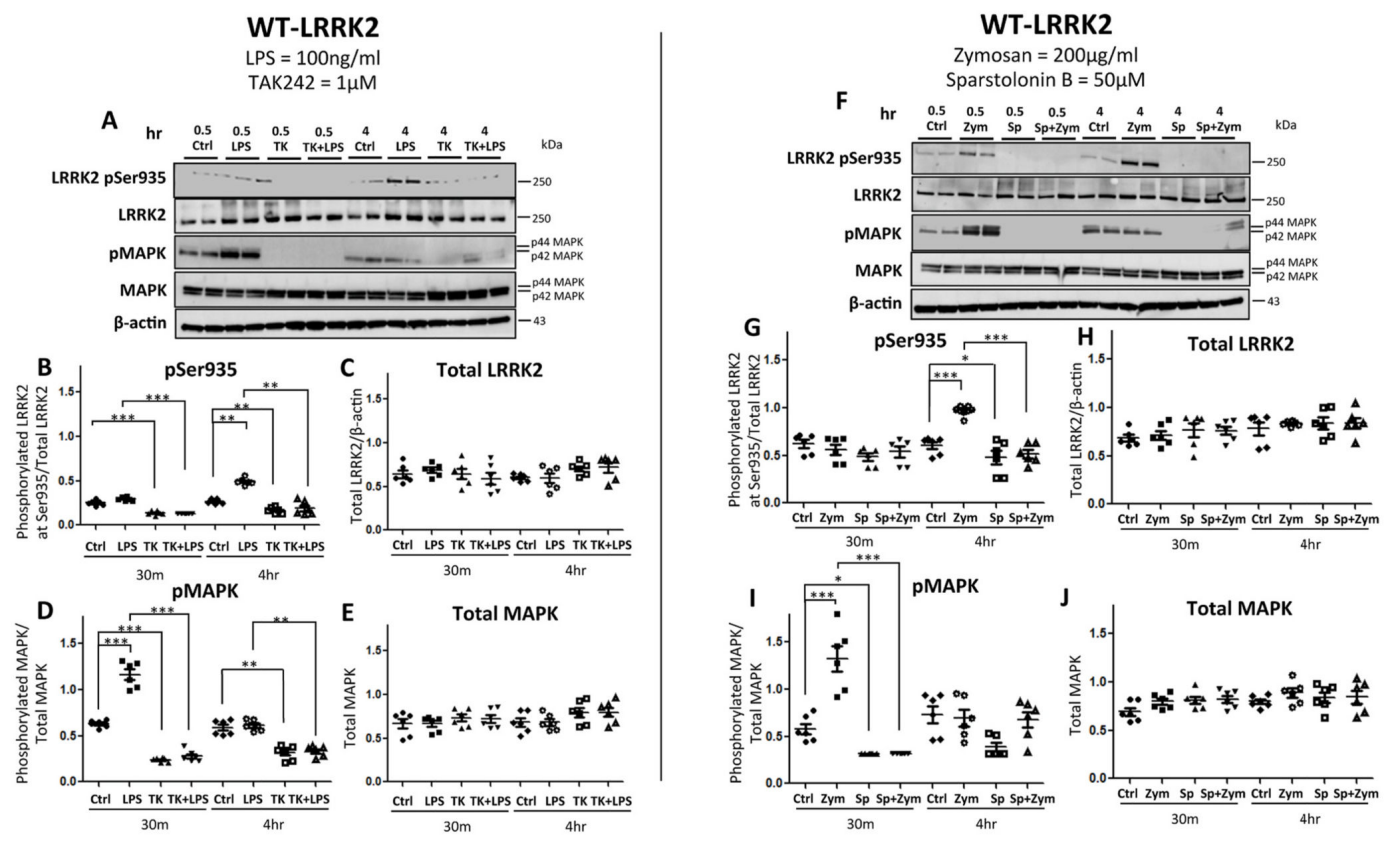
LRRK2 phosphorylation evoked by LPS and zymosan is inhibited with TAK242 and Sparstolonin-B treatments in WT-LRRK2 RAW264.7 macrophage cells: WT-LRRK2 RAW264.7 cells were pre-treated with either TAK242 [TLR4 inhibitor; (1 μM)] or Sparstolonin-B [TLR2 inhibitor; (50 μM)] for 45 min followed by LPS (100 ng/ml) (A) and zymosan (200 μg/ml) (F) treatments for 4h. Cell pellets were collected at 30 min and 4h time points and subjected to immunoblots with the indicated antibodies. Controls contain media only. Blots were probed with LRRK2 phosphorylation antibody Phospho-Ser935, Phospho-p44/p42 MAPK (pMAPK), as well as total LRRK2 and MAPK with LPS (A-E) and zymosan (F-J). Values represent the mean ± S.E.M. of 3 independent experiments (with internal duplicates in each experiment). * and *** signify p < 0.05 and 0.001 respectively. Statistical comparisons carried out by repeated measures one-way ANOVA with Tukey’s posthoc test.

In order to confirm the specificity of the effects of LPS and zymosan on TLR4 and TLR2 respectively, we pre-treated cells with TAK242 (TLR4 antagonist) at 1 μM or Sparstolonin B (TLR2 antagonist) at 50 μM for 45min prior to treatment with LPS (100 ng/ml) or Zymosan (200 μg/ml). Cells were collected at 30min and 4h to measure phosphorylated MAPK (pMAPK) and phosphorylated Ser935 (pSer935). Treatment with TAK242 in WT-LRRK2 and T1348N-LRRK2 cells alone caused suppression of pSer935 and pMAPK at both 30min and 4h (Fig 5B,D and Fig. S5B,D). Sparstolonin B treatment alone had an inhibitory effect on pMAPK and Ser935 phosphorylation (Fig. 5G,I and Fig. S5G,I). LPS or zymosan stimulation induced the expected significant increase in pMAPK at 30min and pSer935 at 4h with TAK242 and Sparstolonin B preventing this in both WT-LRRK2 and T1348N-LRRK2 cells (Fig. 5B,G, Fig. 5D,I, Fig. S5B,G and Fig. S5D,I).

### 3.7 Rab10 phosphorylation is decreased in iPS-Macs following LPS treatment

**Fig. 6:**
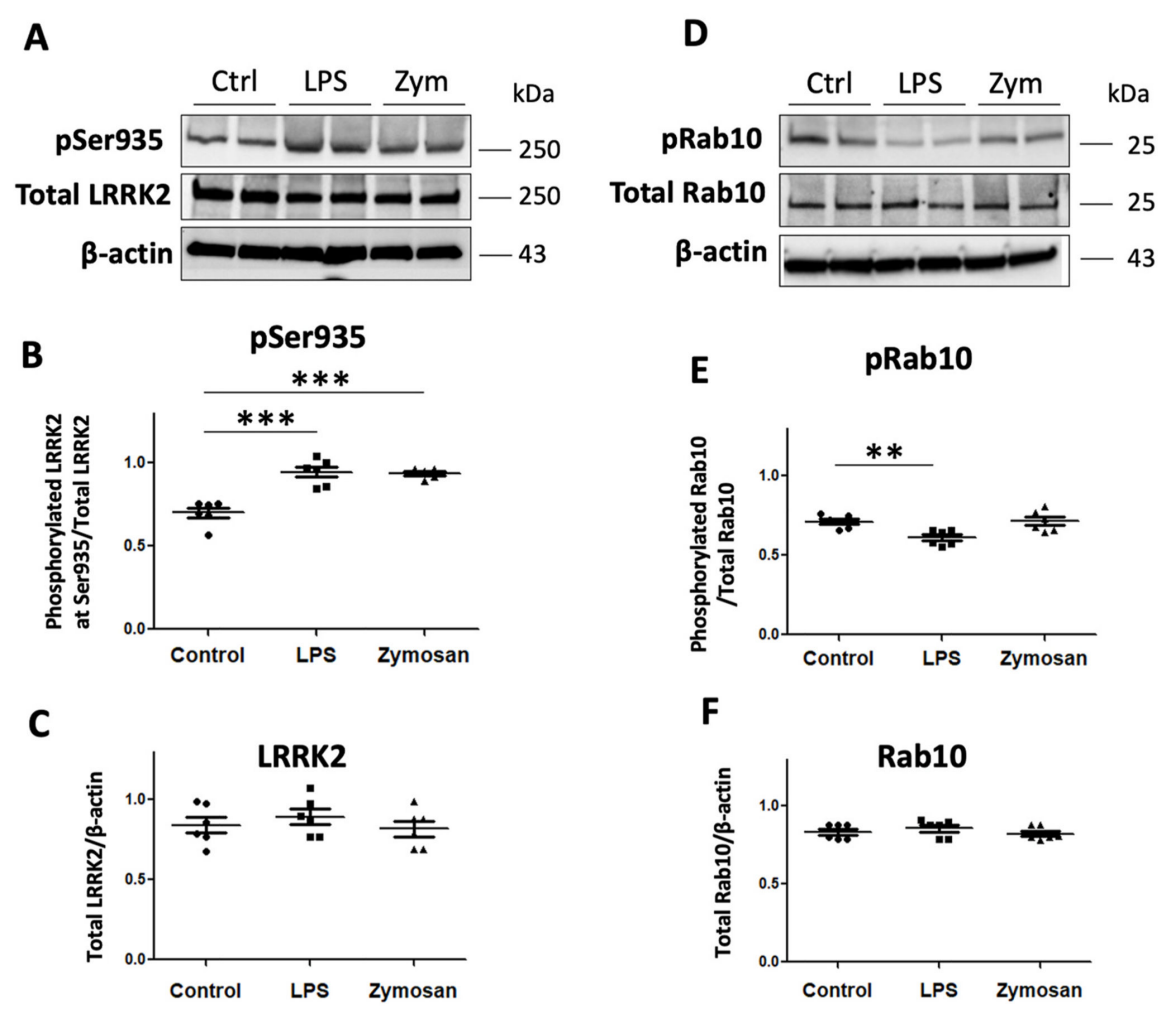
Basal and stimulated levels of phosphorylated Ser935 and phosphorylated Rab10 in WT-LRRK2 RAW264.7 macrophages with LPS and zymosan. Immunoblots from WT-LRRK2 of basal and stimulated with LPS and zymosan phospho-Ser935 levels (A, B) and total LRRK2 levels (A, C). Immunoblots from WT-LRRK2 of basal and stimulated with LPS and zymosan phospho-Rab10 levels (D, E) and total LRRK2 levels (D, F). Values represent the mean ± S.E.M. of 3 independent experiments (with internal duplicates in each experiment). Statistical significance was determined using one-way ANOVA with Tukeys post-hoc test. *** and ** denotes p < 0.001 and p < 0.01 respectively.

In order to examine whether the decrease in Rab10 phosphorylation also occurred in human immune cells, we quantitated the effects of LPS treatment on LRRK2 and Rab10 phosphorylation in iPS-Macs. Firstly we showed a significant increase in Phospho-LRRK2 Ser935 with LPS treatment at 4hs with a concomitant significant decrease in phospho-Rab10 levels compared with non-LPS treated IPS-Macs (Fig. 6). Zymosan treatment had minimal effect on phospho-Rab10 levels in these cells.

### 3.8 Quantitation of mRNA levels of TLR4/TLR2 in WT-LRRK2, T1348N-LRRK2 and KO-LRRK2 cell lines

In order to assess whether the expression levels of TLR4 and TLR2 are similar between the three cell lines, we measured mRNA levels of TLR4 and TLR2 levels by qRT-PCR. Our results demonstrate that there was a small but significant decrease in TLR2 mRNA levels in T1348N-LRRK2 compared with WT-LRRK2 cells (Fig. S6A), whilst a small but significant increase of TLR4 mRNA levels was noted in the T1348N-LRRK2 cell line over WT-LRRK2 (Fig. S6B).

### 3.9 Live/dead assay

In order to test whether the LPS and zymosan doses used in the cell culture experiments are not affecting cell viability, we performed the live/dead assay after treating WT-LRRK2 RAW264.7 cells for 24h. Our results indicate that there were no significant differences in the percentage of live cells between any of the three cell lines, WT-, T1348N- and KO-LRRK2 RAW264.7 cell lines either at basal or following stimulation with LPS or zymosan at the doses used for the duration of 24h (Fig. S7). This indicates that the LPS and zymosan doses used in our experiments are non-toxic to cells, and do not affect cell viability.

### 3.10 Measurement of cytokine release by ELISA of TNFα, IL-6 and IL-10 in WT-LRRK2, T1348N-LRRK2 and KO-LRRK2 cell lines

**Fig. 7:**
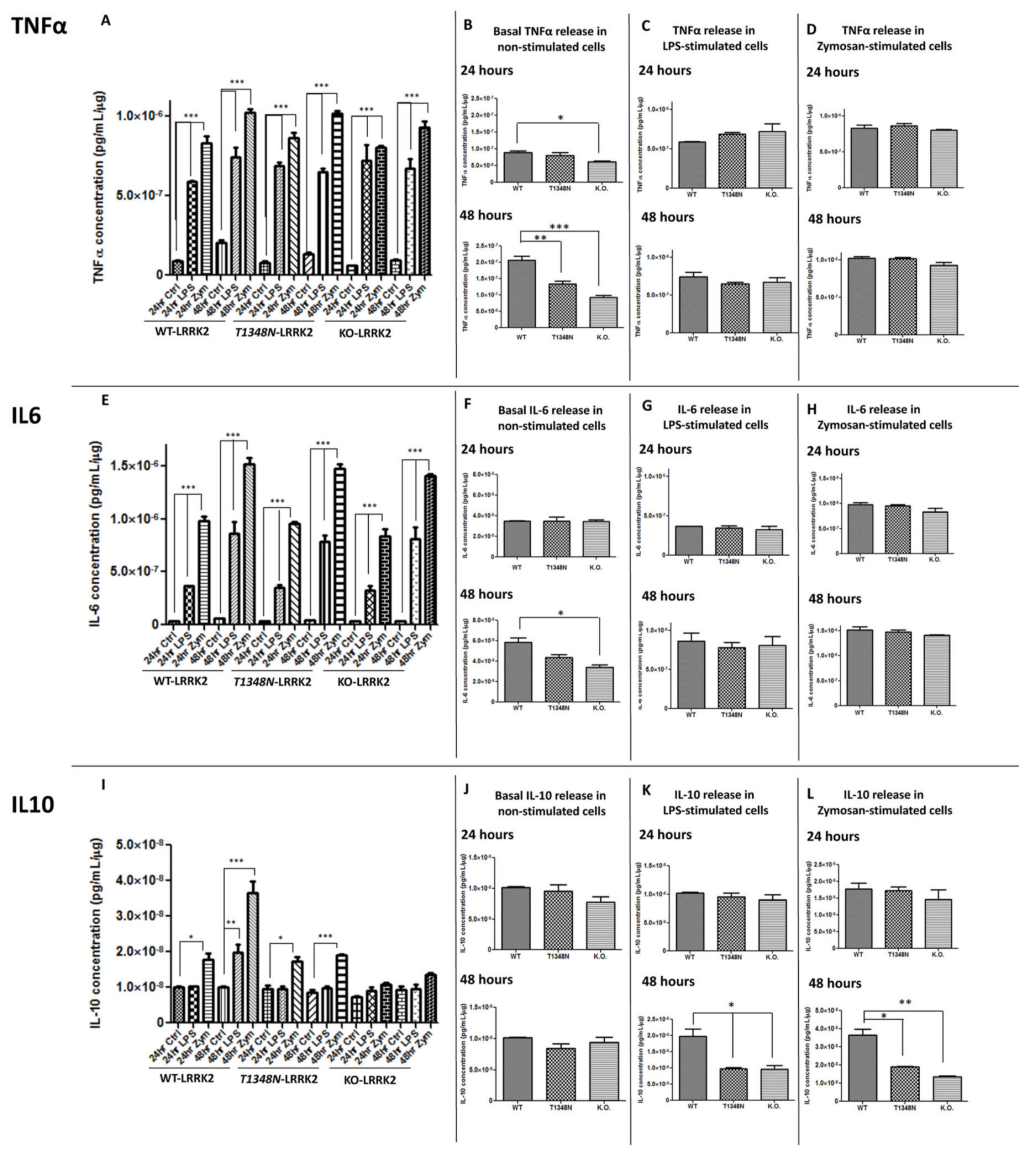
Basal and stimulated TNFα, IL-6 and IL-10 release with LPS and zymosan in WT-LRRK2, T1348N-LRRK2 and KO-LRRK2 RAW264.7 macrophage cells. Basal and stimulated TNFα (A-D), IL-6 (E-H) and IL-10 (I-L) release were measured in WT-LRRK2, T1348N-LRRK2 and KO-LRRK2 cell lines with LPS (100 ng/ml) and zymosan (200 μg/ml) treatment at 24 and 48 h time points. Controls contain media only. Values represent the mean ± S.E.M. of 3 independent experiments (with internal duplicates in each experiment).Statistical comparisons carried out by repeated measures one-way ANOVA with Tukey’s post-hoc test. *** denotes p < 0.001.

In order to test if cytokine release was affected in the three RAW264.7 cell lines at both basal levels and following LPS or zymosan stimulation, we examined the release of TNFα, IL-6 and IL-10 using specific ELISAs. TNFα and IL-6 levels were significantly increased with both LPS and zymosan treatments in the three cell lines at both 24h and 48h with the effect of zymosan seeming to be greater than LPS (Fig 7A, E). The basal levels of release of TNFα and IL-6 measured by ELISA at 24h and 48h showed a significant decrease in TNFα in KO-LRRK2 cell line at 24h (Fig. 7B). Additionally, a significant decrease in the basal level of TNFα was noted in both T1348N-LRRK2 and KO-LRRK2 cell lines at 48h (Fig. 7B). However, a significant decrease in basal level of IL-6 was noted in only KO-LRRK2 cell line at 48h (Fig. 7F). However, no significant differences were noted in released TNFα or IL-6 with LPS or zymosan treatment between the three cell lines at either time point (Fig. 7C,D,G,H).

In contrast, a significant increase in IL-10 release was observed with zymosan treatment in WT-LRRK2 and T1348N-LRRK2 cell lines only at both 24h and 48h. LPS treatment only caused a significant increase in IL-10 in WT-LRRK2 at 48h but not at the earlier time point of 24h. Interestingly, there was no significant release of IL-10 in KO-LRRK2 with either LPS and zymosan at both time points (Fig. 7I). Basal IL-10 release levels remained similar in all of the three cell lines at both 24h and 48h (Fig. 7J). However, there was a significant decrease in released IL-10 in T1348N-LRRK2 and KO-LRRK2 at 48h with both LPS and zymosan treatments (Fig. 7K,L). This is in contrast to TNFα and IL-6 release indicating a possible role of WT-LRRK2 in IL-10 release in RAW264.7 cells.

## 4 Discussion

Evidence from recent research supports an important role for LRRK2 in immune cell function (Wallings and Tansey, 2019). In this study, we have performed a detailed time course analysis of LRRK2, Rab8 and Rab10 phosphorylation in RAW264.7 murine macrophage cells following two different inflammatory stimuli targeting different TLRs. In addition, we have compared the cytokine release between the cell lines at basal levels and when stimulated with inflammatory stimuli. The T1348N-LRRK2 and KO-LRRK2 cells have not been previously characterised in terms of LRRK2 phosphorylation and cytokine release. This is important in validating the role of LRRK2 in immune signalling and how this may contribute to PD pathogenesis.

The steady state phosphorylation of four phospho-sites in LRRK2 (Ser910, Ser935, Ser955, Ser973) and Rab8 and Rab10 phosphorylation levels revealed no detectable LRRK2 or any of the phospho-LRRK2, pRab8 and pRab10 proteins in KO-LRRK2 RAW264.7 cell line. This authenticates the homozygous KO-LRRK2 RAW264.7 cell line and lends further evidence to the notion that Rab8 and Rab10 are indeed bonafide substrates of LRRK2 kinase activity (Steger et al., 2016, 2017). There was a significant reduction of steady state LRRK2 levels in the T1348N-LRRK2 cell line as compared to WT-LRRK2 cell line which is expected due to previous observations (Nguyen and Moore, 2017), again suggestive of the notion that T1348N mutation causes LRRK2 protein to destabilise and degrade faster.

Our data shows that LPS and zymosan treatments affect the phosphorylation of LRRK2 at Ser935 residue to similar extent and is sustained until 24h at least which is similar to the effect previously observed (Dzamko et al., 2012; Reynolds et al., 2014) in both WT-LRRK2 and T1348N-LRRK2 RAW264.7 cell lines. We found a high basal level of phosphorylated Ser910 in both the cell lines tested which did not further increase with either LPS or zymosan stimulation at all the time-points examined, although increased Ser910 phosphorylation was shown by Dzamko et al. (2012) in bone marrow-derived macrophages (BMDMs) and RAW264.7 cells at as less as 30min. Why we did not observe an increase of pSer910 with inflammatory stimulus is a matter of conjecture but it could be that RAW264.7 cell lines have a higher level of basally phosphorylated LRRK2 compared to BMDMs. Quantitation from immunoblots can only be semiquantitative in nature and may not be very sensitive to subtle alterations in protein levels. Moreover, in studies of kinase inhibition, pSer935 is the preferred readout used by several researchers (Doggett et al., 2012; Dzamko et al., 2012; Reynolds et al., 2014). However, both LPS and zymosan treatments increased pSer935 significantly over untreated conditions from 2 to 24h time-points. To our knowledge, the Ser955 and Ser973 residues have not been previously examined after treatment with LPS or zymosan in RAW264.7 cells. Previous studies have shown that pSer955 and pSer973 sites are present in WT-HEK cells and these were sensitive to LRRK2 kinase inhibition (Doggett et al., 2012; Reynolds et al., 2014). Our data shows that both these sites are significantly phosphorylated with both LPS and zymosan in WT-RAW264.7 cell lines. Our data also suggest that T1348N mutation has little additional effect on LRRK2 phosphorylation patterns compared to WT-LRRK2 RAW264.7 cells. Therefore loss of LRRK2 GTP binding due to the T1348N variant may not influence LRRK2 phosphorylation at any of the four phospho-serine residues.

The LRRK2 residues at Ser910 and Ser935 are also constitutive phosphorylation sites of LRRK2 which showed no phosphorylation in the presence of pathogenic mutations of PD (Nichols et al., 2010). The Ser910 and Ser935 residues of LRRK2 are shown to be constitutively phosphorylated by other kinases namely IKK_α_, IKK_β_, IKK_ε_, TBK1 (Dzamko et al., 2012) and also by CK1-α (Chia et al., 2014) whilst PP1A is the phosphatase that is responsible for the dephosphorylation of these sites (Lobbestael et al., 2013; Mamais et al., 2014). Phosphorylation at LRRK2 residues at Ser910/935 LRRK2 is associated with 14–3–3 binding, regulating localisation of LRRK2 and downstream signalling events and can lead to altered cytoplasmic localisation (Nichols et al., 2010). Therefore an increase in pSer935 in our experiments with LPS or zymosan treatment may indicate indirect LRRK2 activation which enhances its capacity to bind 14–3–3 protein and potentially will have downstream signalling implications for a sustained time period of 4h to 24h. In contrast, LRRK2 kinase inhibition causes dephosphorylation of Ser910/935 residues leading to disrupted 14–3–3 binding (Dzamko et al., 2012). In this context, evaluation of endogenous LRRK2Ser1292 phosphorylation as a direct readout for LRRK2 kinase autophosphorylation activity would allow direct comparison of LRRK2 phosphorylation however we were unable to detect this in our experimental system (data not shown). Detection of endogenous phosphorylated Ser1292 is technically challenging, and may require further enrichment procedures (Kluss et al., 2018). The phosphorylation of LRRK2 at these key residues can be very dynamic and previous data from our lab has shown that oxidative stress can disrupt this interaction resulting in dephosphorylation of LRRK2 at the key residues (Mamais et al., 2014).

Pathogenic mutations within the GTPase domain of LRRK2 enhance phosphorylation of Rab isoforms and T1348N mutation has been shown to prevent Rab29 mediated recruitment of LRRK2 to the Golgi and concomitant LRRK2 activation (Purlyte et al., 2018). Therefore, we studied T1348N-LRRK2 in parallel to WT-LRRK2 cells. However, we noted that T1348N-LRRK2 cells also demonstrated similar phosphorylation changes with both LPS and zymosan treatments compared to WT cells for LRRK2 Ser910, Ser935, Ser955 and Ser973, hence, T1348N-LRRK2 depicted a very similar effect on LRRK2 phosphorylation to WT-LRRK2 cells. Our data also indicate that T1348N does not differentially affect the extent of LRRK2 phosphorylation compared to WT-LRRK2 cells upon TLR stimulation.

Furthermore, our RAW264.7 cell types did not show any increase in total LRRK2 levels with either LPS or zymosan treatment. An increase in expression of LRRK2 mRNA and protein in response to IFNγ have been observed in human B- and T cells, BMDMs and primary human microglia (Dzamko et al., 2012). More recently IFNγ treatment increased LRRK2 protein expression in human induced pluripotent stem cells (hiPSC) macrophages and microglia. Contrary to this, our data do not suggest any significant alteration in LRRK2 protein levels in either WT- or T1348N-LRRK2 RAW264.7 cell lines with either LPS or zymosan stimulation. It is likely that the LRRK2 protein levels are altered only with treatment with IFNγ.

Recent studies have shown that Rab proteins are bona fide phosphorylation targets of LRRK2 (Steger et al., 2016, 2017) and these are now being validated and established by various research groups as read-outs for LRRK2 phosphorylation activity in various cell types (Atashrazm et al., 2019; Ito et al., 2016). The link between LRRK2 and Rabs has been shown in a study where mutant LRRK2 impaired late endosomal trafficking via Rab7 function regulation (Gomez-Suaga et al., 2014). The hyperactive LRRK2 mutant was shown to phosphorylate Rab1A, Rab1B, Rab3A, Rab8A, Rab10, Rab12 and Rab29 *in vitro* (Steger et al., 2016, 2017). Rab3A is important for neurotransmission and neurotransmitter exocytosis (Steger et al., 2016) while Rab8 is critical in neurite outgrowth of neurons, vesicular transport and autophagy. Rab10 is associated with ciliogenesis and TLR4 recycling from endo-somes/Golgi to the plasma membrane (Banton et al., 2014; Homma and Fukuda, 2016; Peranen et al., 1996; Rivero-Rios et al., 2019) and dysregulation of these highly vital cellular activities could provide a mechanism leading to differential neuronal vulnerability, dopaminergic cell death, and hence PD. LRRK2 induced phosphorylation of Rab10 inhibits its function by preventing binding to Rab-GDP dissociation inhibitor factors necessary for membrane delivery and recycling and this may impair autophagic function (Steger et al., 2016). Malfunction of autophagy may account for early accumulation of phospho-Ser129-α-synuclein, a marker of PD pathology, which is normally degraded by autophagy (Di Maio et al., 2018) and is characteristic of PD pathology. Using Phos-tag analysis, it has been shown recently that LRRK2 inhibitors markedly dephosphorylate Rab10 within minutes and more rapidly than LRRK2 Ser935/Ser1292 biomarker sites (Ito et al., 2016). However, only a very small proportion of Rab proteins are phosphorylated at any one time (~1%) (Ito et al., 2016; Steger et al., 2016). Relevant to pathophysiology of PD, Rab10 was shown to be phosphorylated in human neutrophils and was sensitive to a specific LRRK2 kinase inhibitor (Fan et al., 2018). Hence, in our study we particularly investigated Rab8 and Rab10 as direct substrates of LRRK2 after stimulation with LPS and zymosan, TLR4 and TLR2 agonists respectively.

A key finding of our investigations is that Rab10 phosphorylation was decreased with LPS stimulation but not with zymosan in both WT- and T1348N-LRRK2 RAW264.7 cell lines and in human iPS-Macs. In contrast, Rab8 phosphorylation remained unchanged with LPS and zymosan treatments in the Raw264.7 cell lines, a novel observation. Our data suggests that triggering immune signalling through TLR4 stimulation can affect LRRK2 functioning through either stimulating dephosphorylation of Rab proteins or could lead to a decrease in LRRK2 kinase activity caused by LPS; the second scenario is a matter of debate as LPS treatment stimulated LRRK2 phosphorylation but whether this is a direct or indirect stimulation by other LRRK2 kinases remains a matter of conjecture. Previous work has shown that LRRK2 kinase inhibitors did not have an affect on LPS-stimulated phosphorylation of LRRK2 (Dzamko et al., 2012) whilst a recent publication by Xu and co-workers have shown that IFNγ treatment increases Rab10 phosphorylation and that TAK242 treatment did not attenuate this effect (Xu et al., 2020). Intriguingly, another recent study has shown that overexpression of WT-PPM1H ablated the phosphorylation of Rab10 following overexpression of R1441G-LRRK2 and catalytically inactive PPM1H failed to induce dephosphorylation of Rab10 (Berndsen et al., 2019). Although our data suggests no alteration of PPM1H levels, we cannot rule out an increase in its phosphatase activity – this aspect remains to be investigated and is beyond the scope of the current study. Moreover, a recent study has shown that Rab8 and Rab10 are recruited to mature phagosomes which is LRRK2 dependent (Lee et al., 2020). Immune signalling and lysosomal stress both induce translocation of LRRK2 and its phospho-substrates Rab8a and Rab10 on to stressed lysosomes in different cell types (Eguchi et al., 2018). Interestingly Rab8a phosphorylation is increased by all pathogenic LRRK2 mutations (Mamais et al., 2020). However, it is of interest that decreased Rab10 phosphorylation occurred only with LPS stimulation and not with zymosan treatment, suggesting that this effect is sensitive to TLR4 stimulation.

One pathway through which TLR4 stimulation activates mitogen-activated protein kinase (MAPK) pathway is via TRAF6, which recruits receptor-interacting protein kinase (RIPK) proteins leading to phosphorylation of MAPK (Dainichi et al., 2019) which is an early event in the signalling cascade. Our data show MAPK phosphorylation peaking at the earlier time-point of 30min but returning to control levels at 4h and this effect was consistent in both WT-LRRK2 and T1348N-LRRK2 cell lines. The phosphorylation of LRRK2 measured for Ser935 site occurred later from 2h onwards similar to what has been observed in an earlier study using bone-marrow derived macrophages (Dzamko et al., 2012). Furthermore, The KO-LRRK2 cell line also followed the same pattern of MAPK phosphorylation suggesting that TLR4 signalling is comparable to the other two cell lines where LRRK2 protein is present. The data also suggest that LPS and zymosan-induced LRRK2 phosphorylation is a secondary effect in TLR signalling and that T1348N-LRRK2 mutation may not play a part in modulating MAPK phosphorylation any further. Collectively our data show that MAPK phosphorylation is an earlier event in LPS-induced signalling pathways even when LRRK2 is absent. Further research is now needed to delineate the precise molecular events linking LPS and zymosan to LRRK2 phosphorylation.

In our study, we used TAK242 (small molecule inhibitor for TLR4 signalling) and Sparstolonin B (TLR2 antagonist) to study the specific effects of LPS/TLR4 and zymosan/TLR2 on MAPK and LRRK2 Ser935 phopshorylation. We demonstrate that TAK242 causes a significant downregulation of pMAPK and LRRK2 pSer935 in both WT-LRRK2 and T1348N-LRRK2 cell lines. In contrast, Sparstolonin B did not show downregulation effect to the same extent. Therefore, we have shown that the effect of TAK242 is more sustained on MAPK and LRRK2 in RAW264.7 cell lines. However we also noted that TAK242 and Sparstolonin B treatment on its own demonstrated significant downregulation of MAPK and Ser935LRRK2 phosphorylation which is most likely owing to trace contamination of endotoxin levels in heat-inactivated FBS (as stated in Thermofisher product documentation). TAK242 binds to the intracellular domain of TLR4 which inhibits TLR4 signalling by disrupting the interaction of TLR4 with its downstream adaptor molecules (Matsunaga et al., 2011; Takashima et al., 2009). These data further authenticate MAPK signalling cascade as a downstream effect of TLR receptor stimulation and that this pathway is stimulated prior to LRRK2 phosphorylation.

LRRK2 plays distinct roles in microglia and macrophages and LRRK2 expression is stringently regulated in both peripheral and innate immunity (Lee et al., 2017). It has been shown that in primary macrophages from R1441G, G2019S or LRRK2 knockout mice, there were alterations in LPS-driven cytokine release compared to wild type (Dzamko et al., 2012; Hakimi et al., 2011; Wandu et al., 2015). Our study has investigated TNFα, IL-6 and IL-10 in RAW264.7 cell lines where we observed a significant increase in TNFα and IL-6 secretion in WT-LRRK2, T1348N-LRRK2 and KO-LRRK2 cell lines at both 24h and 48h. However, zymosan seems to have a stronger effect on this secretion compared with LPS at the concentrations of TLR activators we used. Since the levels of secretion of the cytokines with zymosan across these three lines were not significantly different, this indicates that T1348N-LRRK2 and KO-LRRK2 do not significantly influence TNFα and IL-6 secretion. Although it is noteworthy that at basal levels, T1348N-LRRK2 and KO-LRRK2 display reduced TNFα and IL-6 release. Interestingly, we see disrupted secretion of the neuroprotective cytokine IL-10 in both T1348N-LRRK2 and KO-LRRK2. Although we observed subtle changes in TLR2/4 mRNA levels between T1348N and WT-LRRK2 cell lines, it is unlikely that these would result in the striking alterations in IL-10 release observed in both T1348N and LRRK2-KO cell lines. Further investigations are needed to answer these observations. Nevertheless, our data suggests a neuroprotective role of LRRK2 in immune signalling through altered IL-10 secretion and the mechanisms involved should be explored further.

In animal models, loss of LRRK2 decreases pro-inflammatory myeloid cells in brains of rats and decreases neurodegenerative responses to LPS and α-synuclein (Daher et al., 2014).

Although LRRK2 knockdown or kinase inhibition in primary microglia has shown a decrease in the production of pro-inflammatory cytokines TNFα and IL-1β (Russo et al., 2015), there are some other reports which show no change in cytokine release with LRRK2 knockout in BMDMs (Dzamko et al., 2012). The significantly lower TNF-alpha release at basal levels in T1348N and KO cells may indicate that these cells are less inflammatory in nature compared to WT cell lines. Whilst a significant decrease in IL-10 release with LPS and zymosan in T1348N and KO cells may suggest that these cells are capable of modulating pro-inflammatory responses. Our study did not show a disrupted response with TNFα and IL-6 with LRRK2-KO and T1348N-LRRK2 mutations in RAW264.7 cell line with inflammatory stimuli, indicating that the responses are dependent on the specific cell models used for experimentation.

We appreciate that there are some general issues concerning established cell lines that include comparability between various laboratories, their characteristics and stability over time. However, it has been demonstrated that the phenotypic and functional characteristics of RAW264.7 cell line remain stable for up to 30 passages (Taciak et al., 2018). We have carefully planned all our experiments within 6 passage cycles so as to keep the phenotypic variability at a minimum. Moreover, Raw cell line is considered a good model for inflammation and immune functions (Maurya et al., 2013). Additionally we have validated Rab10 dephosphorylation in human iPS-Macro with LPS giving us confidence on the data obtained from Raw cell lines.

**Fig. 8:**
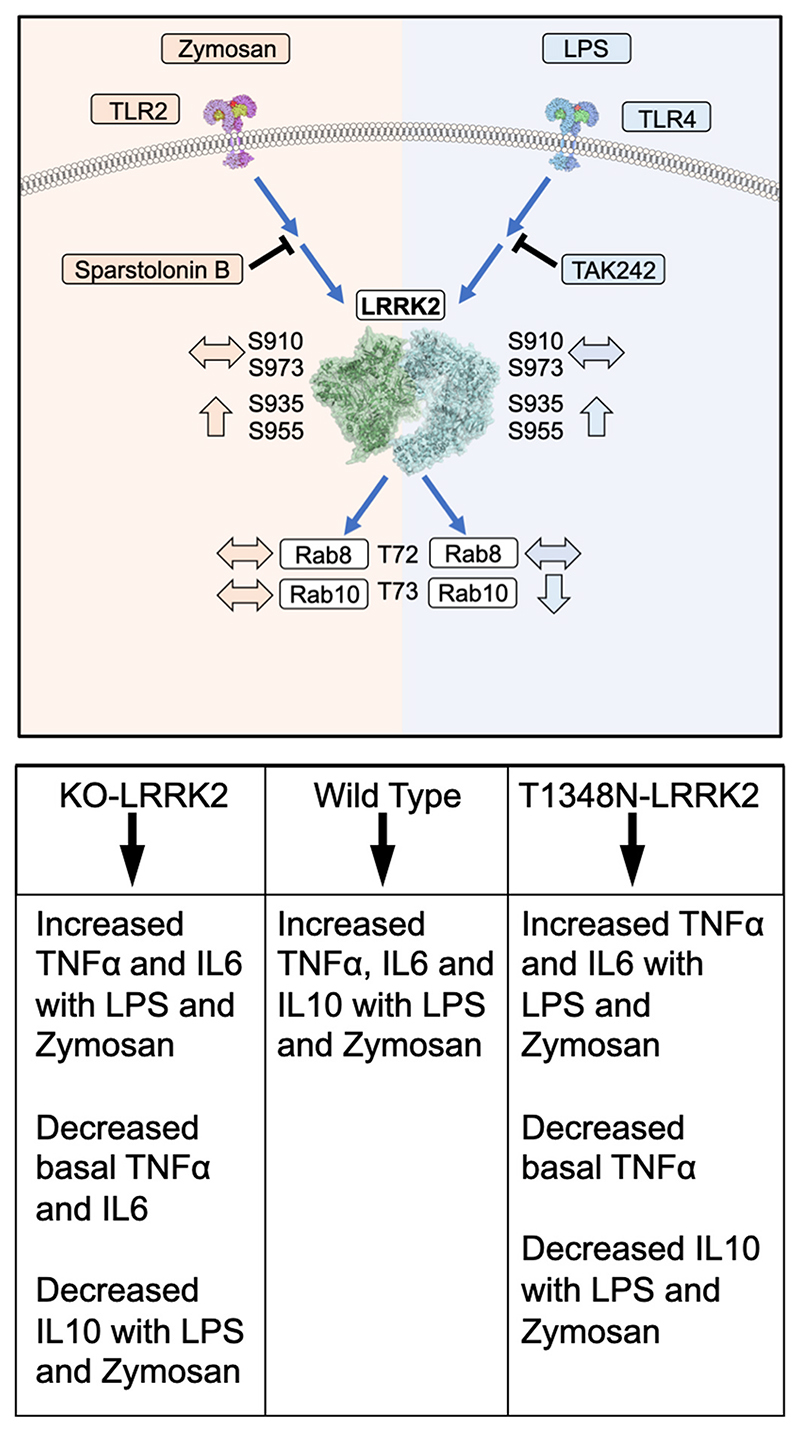
Diagram showing the summary sequence of events in LRRK2 and Rab8/ 10 phosphorylation (top box) and cytokine release (Bottom box) with TLR2 and TLR4 stimulation observed.

In summary, the key and novel finding from our study is that Rab10 phosphorylation is sensitive to TLR4 stimulation and is similarly decreased in both the WT- and T1348N-RAW264.7 cell lines (Fig. 8) and corroborated this in human iPS-Macs. We propose that LPS has an inhibitory effect on LRRK2 kinase activity probably by an indirect mechanism. This also emphasises the importance of Rab proteins being authentic phosphosubstrates of LRRK2. Whilst Ser935/Ser910 are phosphorylated by kinases other than LRRK2, these sites may not be a reliable indicator of LRRK2 kinase activity. Rab8 phosphorylation remained unchanged with either LPS or zymosan stimulation. We also show that pSer955 and pSer973 LRRK2 sites are sensitive to inflammatory stimuli and a significant upregulation is observed in both the WT and T1348N RAW264.7 cell lines. In addition, we show that IL-10 release is altered in KO-LRRK2 and T1348N cell lines with TLR stimulation indicating that WT-LRRK2 influences the release of certain cytokines and these could be context dependent. Interestingly, peripheral inflammatory markers were shown to be elevated in a proportion of asymptomatic PD patients compared to idiopathic PD (Dzamko et al., 2016) and recently it has been shown that innate immunity is important for extracellular alphα-synuclein uptake and degradation (Kim et al., 2021). It is important to note that all of our work has been done in cells expressing endogenous amounts of LRRK2 therefore eliminating confounding factors associated with overexpression paradigms. Our collective data enhances our understanding of the role of LRRK2 in immune cell function and is clearly important in the context of LRRK2-based therapies including small molecule kinase inhibitors and antisense technology which are at various stages of clinical trials (Ahmadi Rastegar and Dzamko, 2020; Padmanabhan et al., 2020).

## Supplementary Material

Fig S1-S7

## Figures and Tables

**Table 1:** Details of antibodies used.

Antibody	Species	Dilution for Western blot	Company	Catalogue numbers
LRRK2-Ser(935)	Rabbit	1:1000	Abcam	133450
LRRK2-Ser(910)	Rabbit	1:1000	Abcam	133449
LRRK2-Ser(955)	Rabbit	1:1000	Abcam	169521
LRRK2-Ser(973)	Rabbit	1:1000	Abcam	181364
Total LRRK2	Rabbit	1:10000	Abcam	133474
PPM1H	Rabbit	1:1000	Invitrogen	PA5–26102
Phospho-Rab8 (T72)	Rabbit	1:1000	Abcam	230260
Phospho-Rab10 (T73)	Rabbit	1:1000	Abcam	230261
Total Rab8	Rabbit	1:1000	Abcam	188574
Total Rab10	Mouse	1:1000	Abcam	104859
Phospho-P44/42	Rabbit	1:1000	Cell Signalling	9101
MAPK (ERK 1/2)			Technology	
P44/42 MAPK (ERK 1/2)	Rabbit	1:1000	Cell Signalling Technology	9102
β-actin	Mouse	1:15000	Sigma Aldrich	A2228
