# Supplementary material for: Abrogation of LRRK2 dependent Rab10 phosphorylation with TLR4 activation and alterations in evoked cytokine release in immune cells
Source: Neurochem Int. Author manuscript; Available in PMC 2021 Jul 1. (PMC7610942; doi:10.1016/j.neuint.2021.105070)
Supplement: Fig S1-S7 [file EMS125897-supplement-Fig_S1_S7.pdf]

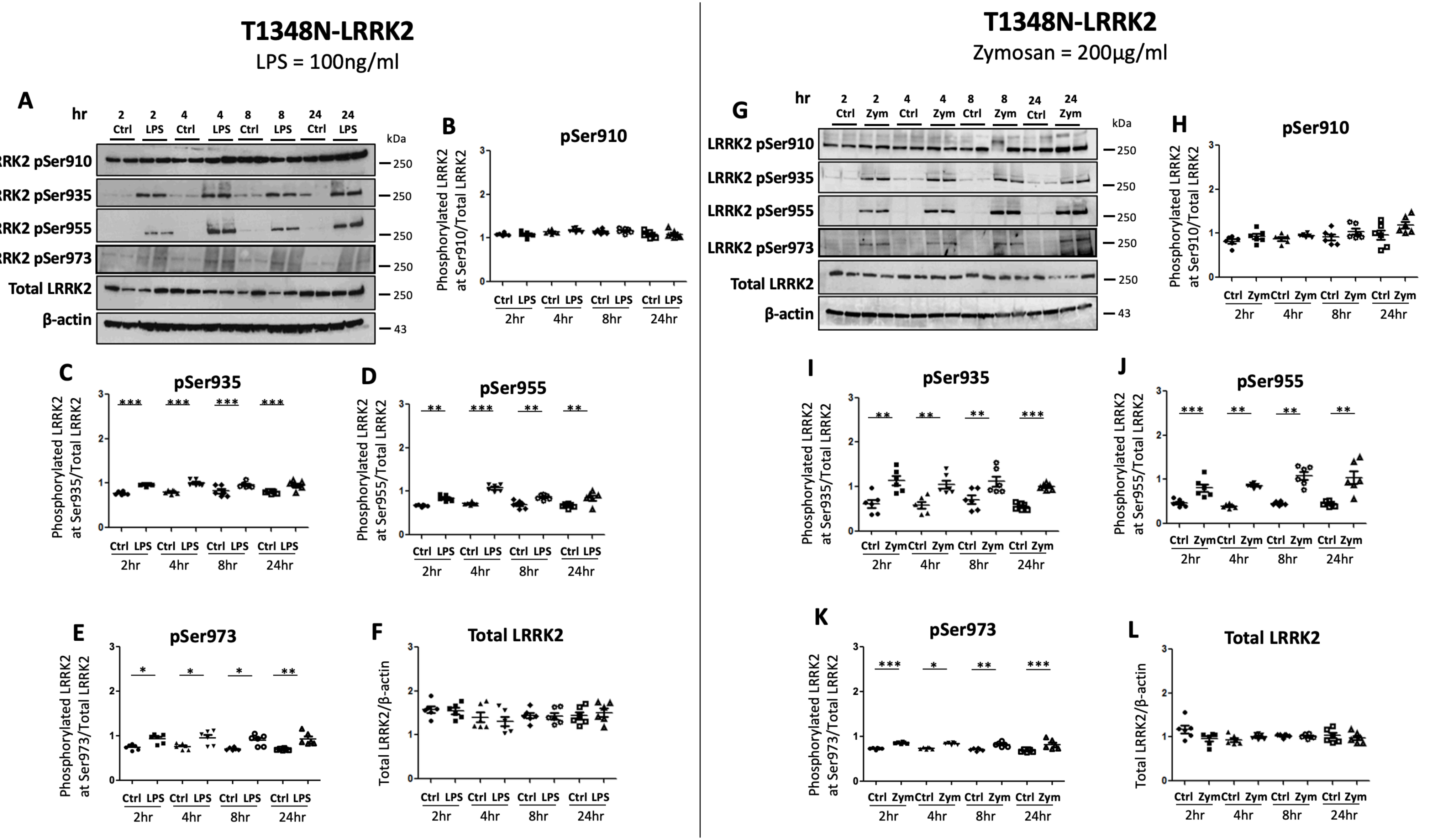
T1348N-LRRK2
LPS = 100ng/ml
A
hr
2 Ctrl
2 LPS
4 Ctrl
4 LPS
8 Ctrl
8 LPS
24 Ctrl
24 LPS
kDa
LRRK2 pSer910
LRRK2 pSer935
LRRK2 pSer955
LRRK2 pSer973
Total LRRK2
β-actin
250
43
B
pSer910
Phosphorylated LRRK2 at Ser910/Total LRRK2
C
pSer935
Phosphorylated LRRK2 at Ser935/Total LRRK2
D
pSer955
Phosphorylated LRRK2 at Ser955/Total LRRK2
E
pSer973
Phosphorylated LRRK2 at Ser973/Total LRRK2
F
Total LRRK2
Total LRRK2/β-actin
Ctrl LPS
2hr 4hr 8hr 24hr
T1348N-LRRK2
Zymosan = 200µg/ml
G
2 Ctrl
2 Zym
4 Ctrl
4 Zym
8 Ctrl
8 Zym
24 Ctrl
24 Zym
H
pSer910
I
pSer935
J
pSer955
K
pSer973
L
Total LRRK2
Ctrl Zym

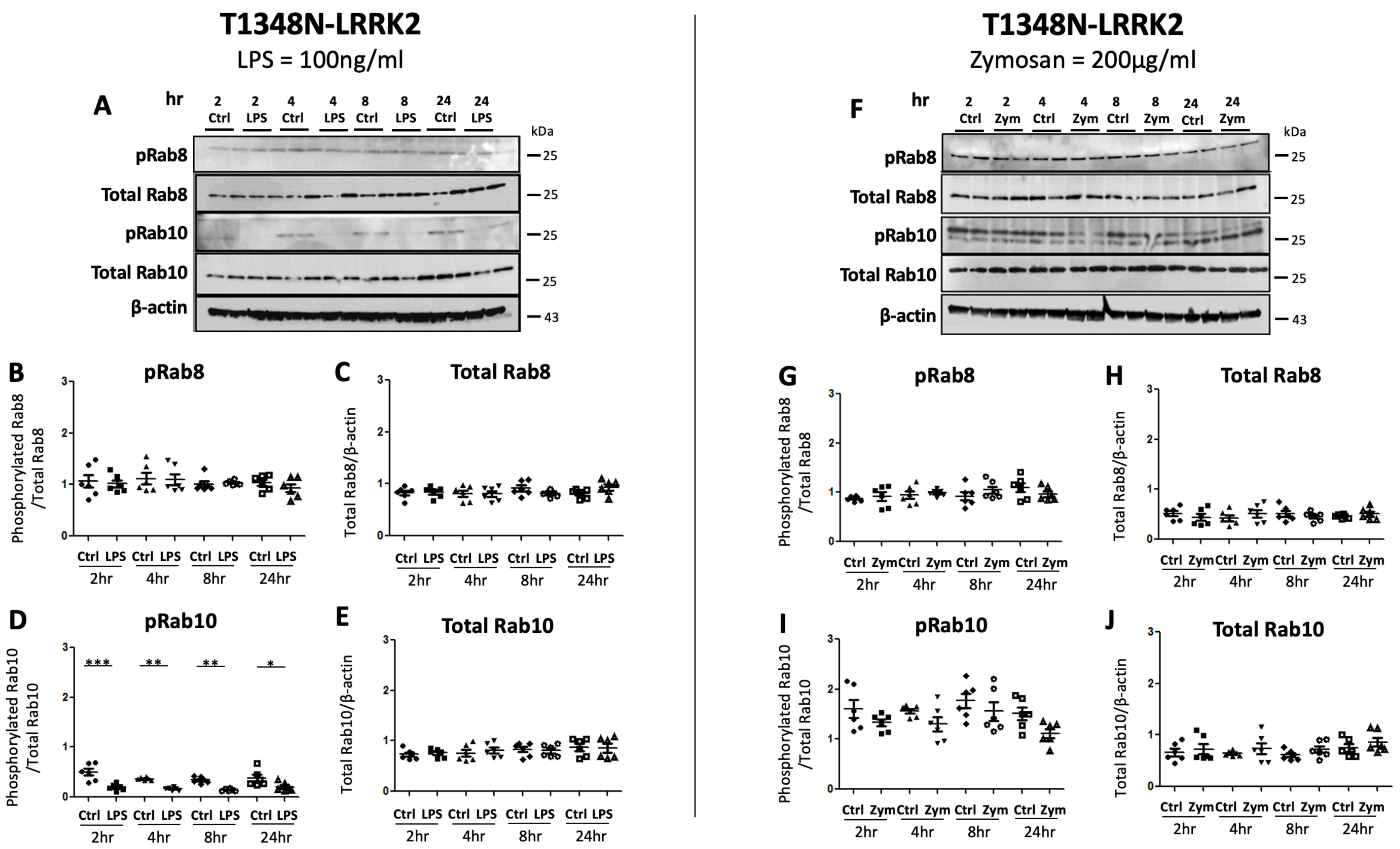
T1348N-LRRK2
LPS = 100ng/ml
A
hr
2 Ctrl
2 LPS
4 Ctrl
4 LPS
8 Ctrl
8 LPS
24 Ctrl
24 LPS
kDa
pRab8
Total Rab8
pRab10
Total Rab10
β-actin
25
25
25
25
43
B
pRab8
Phosphorylated Rab8 /Total Rab8
C
Total Rab8
Total Rab8/β-actin
D
pRab10
Phosphorylated Rab10 /Total Rab10
***
**
**
*
E
Total Rab10
Total Rab10/β-actin
Ctrl LPS
2hr
4hr
8hr
24hr
T1348N-LRRK2
Zymosan = 200µg/ml
F
hr
2 Ctrl
2 Zym
4 Ctrl
4 Zym
8 Ctrl
8 Zym
24 Ctrl
24 Zym
kDa
pRab8
Total Rab8
pRab10
Total Rab10
β-actin
G
pRab8
Phosphorylated Rab8 /Total Rab8
H
Total Rab8
Total Rab8/β-actin
I
pRab10
Phosphorylated Rab10 /Total Rab10
J
Total Rab10
Total Rab10/β-actin
Ctrl Zym

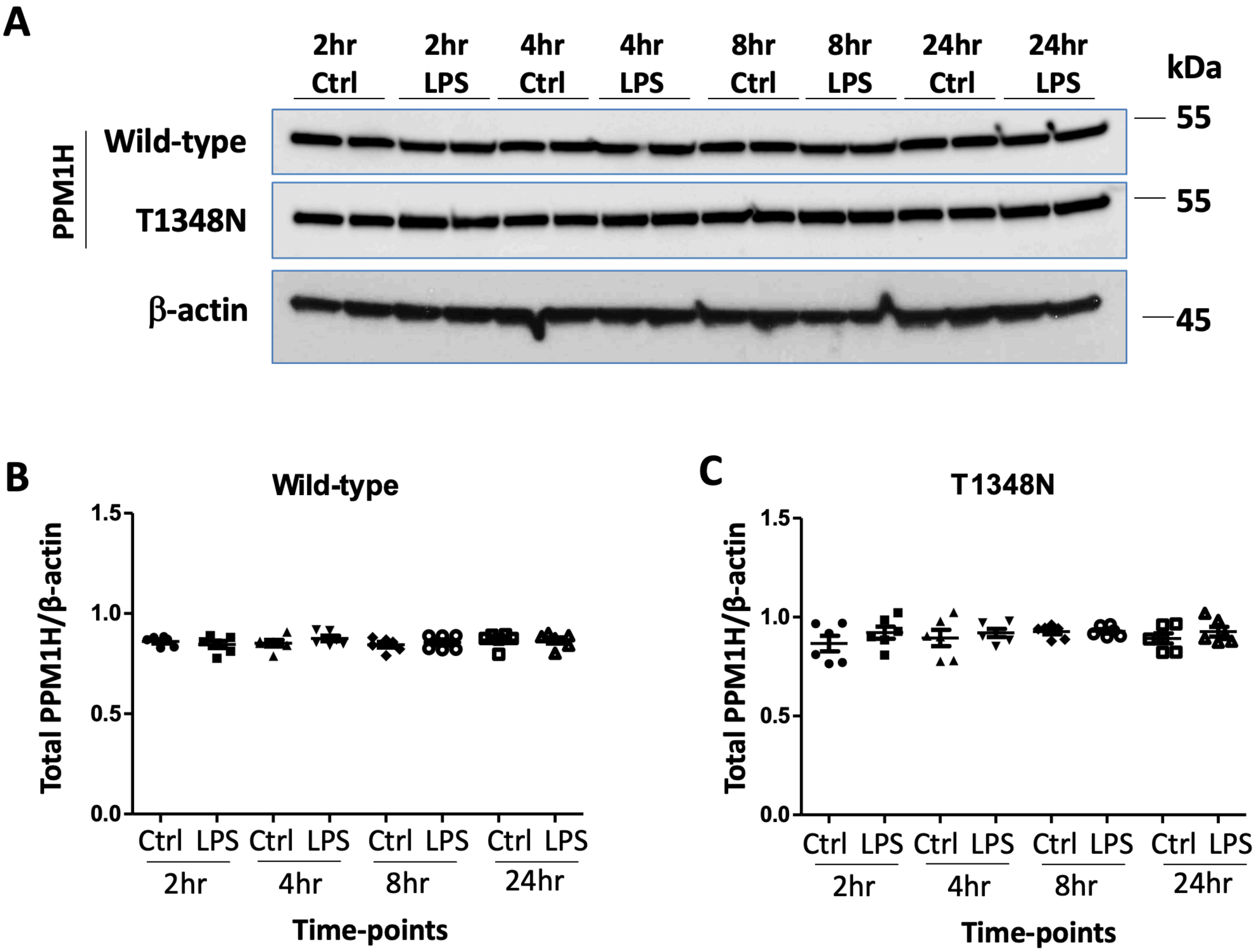
A
2hr Ctrl
2hr LPS
4hr Ctrl
4hr LPS
8hr Ctrl
8hr LPS
24hr Ctrl
24hr LPS
kDa
PPM1H
Wild-type
T1348N
β-actin
55
55
45
B
Wild-type
Total PPM1H/β-actin
1.5
1.0
0.5
0.0
Ctrl LPS Ctrl LPS Ctrl LPS Ctrl LPS
2hr 4hr 8hr 24hr
Time-points
C
T1348N
Total PPM1H/β-actin
1.5
1.0
0.5
0.0
Ctrl LPS Ctrl LPS Ctrl LPS Ctrl LPS
2hr 4hr 8hr 24hr
Time-points

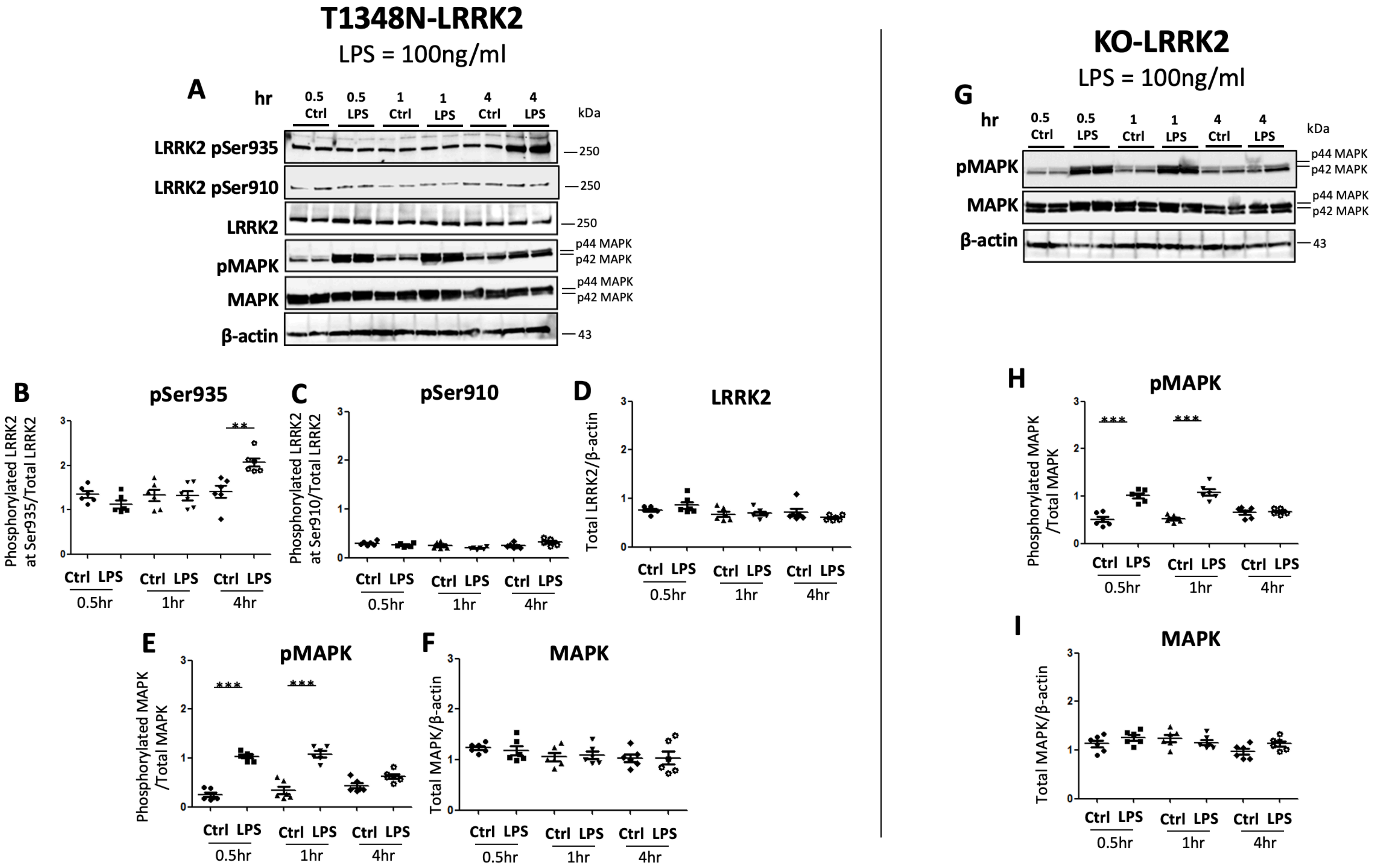
T1348N-LRRK2
LPS = 100ng/ml
A
hr 0.5 Ctrl 0.5 LPS 1 Ctrl 1 LPS 4 Ctrl 4 LPS
kDa
LRRK2 pSer935
LRRK2 pSer910
LRRK2
pMAPK
MAPK
β-actin
250
p44 MAPK
p42 MAPK
43
B
pSer935
Phosphorylated LRRK2 at Ser935/Total LRRK2
C
pSer910
Phosphorylated LRRK2 at Ser910/Total LRRK2
D
LRRK2
Total LRRK2/β-actin
E
pMAPK
Phosphorylated MAPK /Total MAPK
F
MAPK
Total MAPK/β-actin
Ctrl LPS
0.5hr
1hr
4hr
KO-LRRK2
LPS = 100ng/ml
G
H
pMAPK
I
MAPK

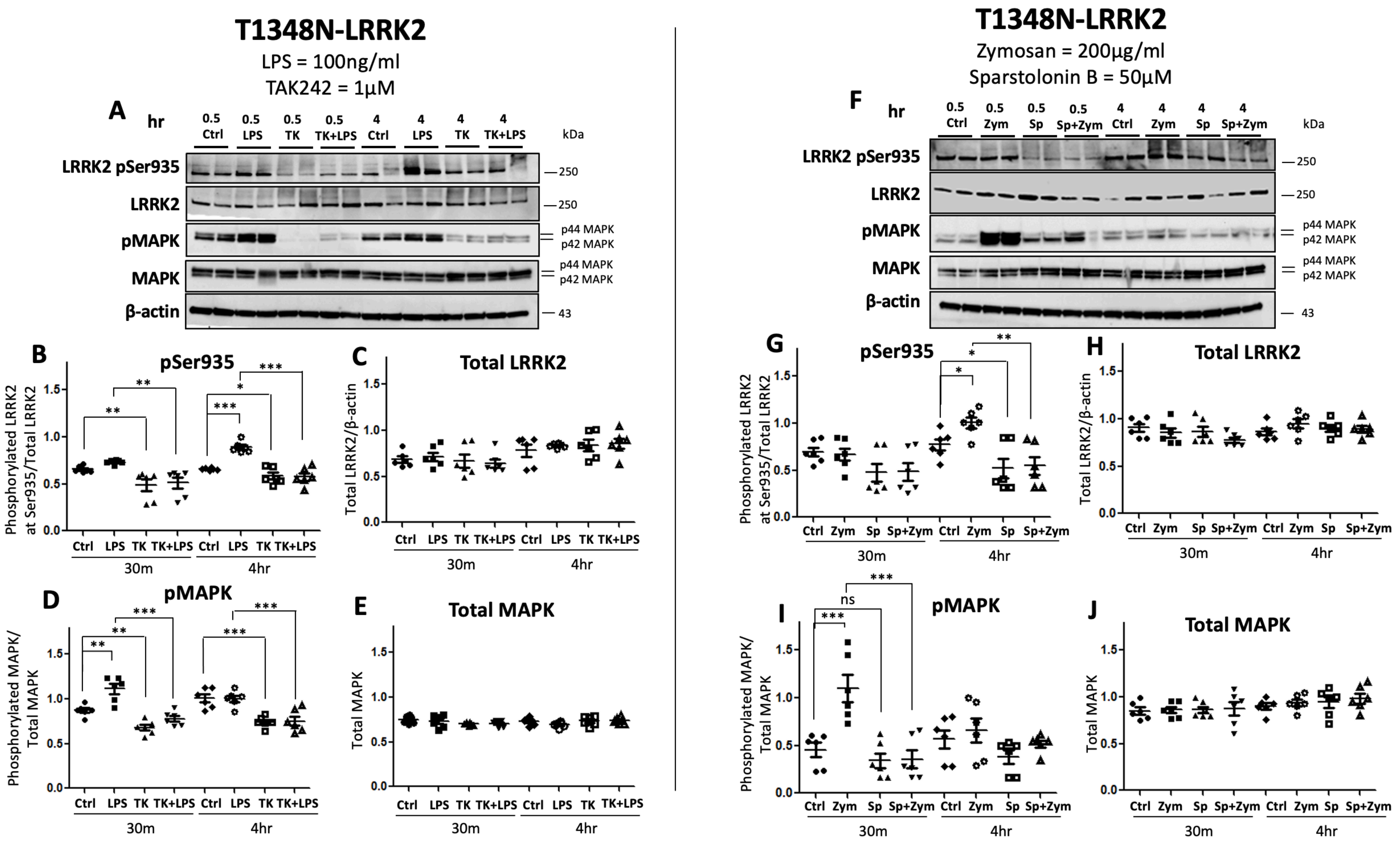
T1348N-LRRK2
LPS = 100ng/ml
TAK242 = 1µM
A
hr
0.5 Ctrl
0.5 LPS
0.5 TK
0.5 TK+LPS
4 Ctrl
4 LPS
4 TK
4 TK+LPS
kDa
LRRK2 pSer935
LRRK2
pMAPK
MAPK
β-actin
250
250
p44 MAPK
p42 MAPK
p44 MAPK
p42 MAPK
43
B
pSer935
Phosphorylated LRRK2 at Ser935/Total LRRK2
C
Total LRRK2
Total LRRK2/β-actin
D
pMAPK
Phosphorylated MAPK/ Total MAPK
E
Total MAPK
Total MAPK
Ctrl LPS TK TK+LPS Ctrl LPS TK TK+LPS
30m
4hr
T1348N-LRRK2
Zymosan = 200µg/ml
Sparstolonin B = 50µM
F
0.5 Ctrl
0.5 Zym
0.5 Sp
0.5 Sp+Zym
4 Ctrl
4 Zym
4 Sp
4 Sp+Zym
G
pSer935
H
Total LRRK2
I
pMAPK
ns
J
Total MAPK
Ctrl Zym Sp Sp+Zym Ctrl Zym Sp Sp+Zym

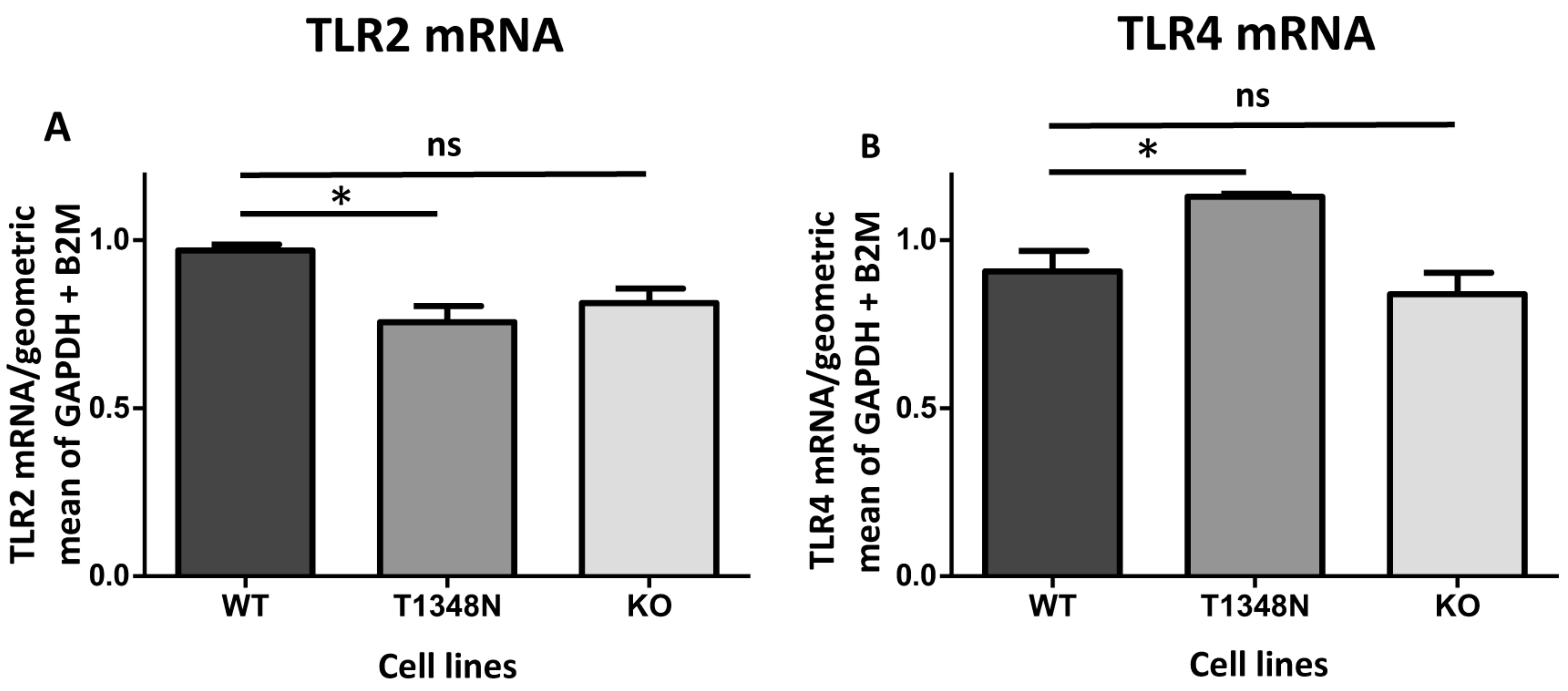
TLR2 mRNA
A
ns
*
TLR2 mRNA/geometric mean of GAPDH + B2M
1.0
0.5
0.0
WT
T1348N
KO
Cell lines
TLR4 mRNA
B
ns
*
TLR4 mRNA/geometric mean of GAPDH + B2M
1.0
0.5
0.0
WT
T1348N
KO
Cell lines

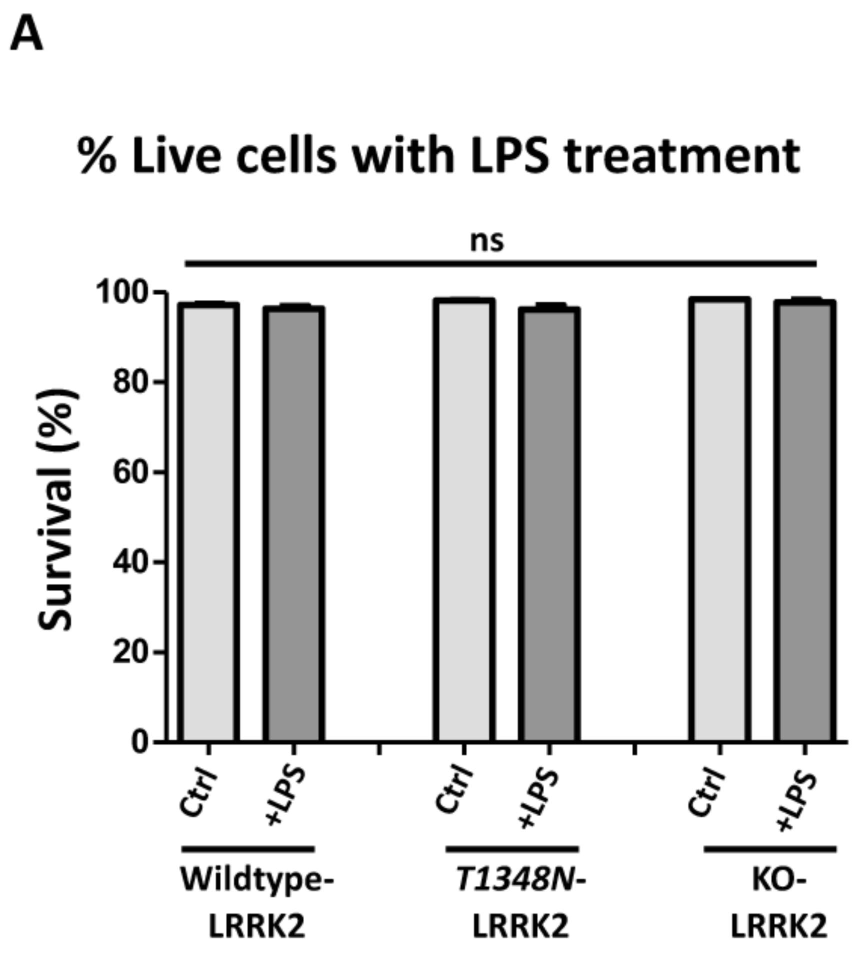
A
% Live cells with LPS treatment
ns
Survival (%)
100
80
60
40
20
0
Ctrl
+LPS
Ctrl
+LPS
Ctrl
+LPS
Wildtype-LRRK2
T1348N-LRRK2
KO-LRRK2

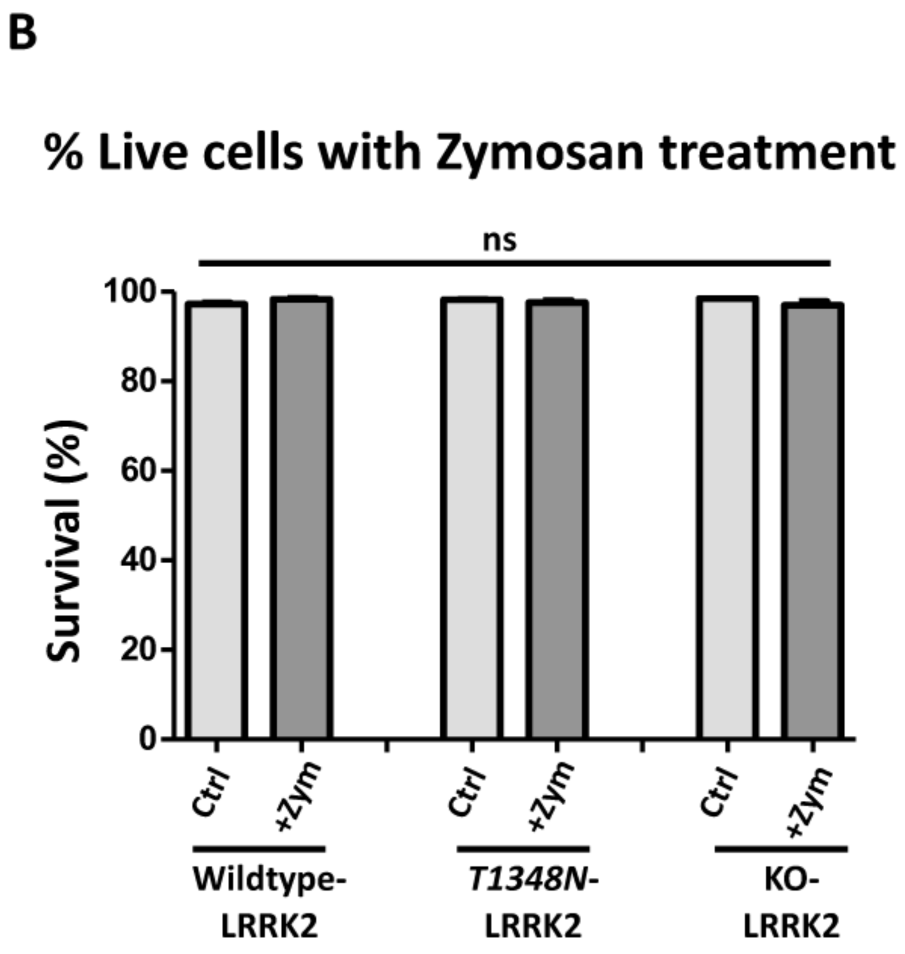
B
% Live cells with Zymosan treatment
ns
Survival (%)
100
80
60
40
20
0
Ctrl
+Zym
Ctrl
+Zym
Ctrl
+Zym
Wildtype-LRRK2
T1348N-LRRK2
KO-LRRK2
